# RSPO2 and RANKL signal through LGR4 to regulate osteoclastic premetastatic niche formation and bone metastasis

**DOI:** 10.1172/JCI144579

**Published:** 2022-01-18

**Authors:** Zhiying Yue, Xin Niu, Zengjin Yuan, Qin Qin, Wenhao Jiang, Liang He, Jingduo Gao, Yi Ding, Yanxi Liu, Ziwei Xu, Zhenxi Li, Zhengfeng Yang, Rong Li, Xiwen Xue, Yankun Gao, Fei Yue, Xiang H.-F. Zhang, Guohong Hu, Yi Wang, Yi Li, Geng Chen, Stefan Siwko, Alison Gartland, Ning Wang, Jianru Xiao, Mingyao Liu, Jian Luo

**Affiliations:** 1 Shanghai Key Laboratory of Regulatory Biology, Institute of Biomedical Sciences and School of Life Sciences, East China Normal University, Shanghai, China.; 2 Precision Research Center for Refractory Diseases, Institute for Clinical Research, Shanghai General Hospital, Shanghai Jiao Tong University School of Medicine, Shanghai, China.; 3 Yangzhi Rehabilitation Hospital (Shanghai Sunshine Rehabilitation Center), Tongji University School of Medicine, Shanghai, China.; 4 Department of Orthopaedic Oncology, Changzheng Hospital, Second Military Medical University, Shanghai, China.; 5 State Key Laboratory of Proteomics, Beijing Proteome Research Center, National Center for Protein Sciences (Beijing), Beijing Institute of Lifeomics, Beijing, China.; 6 Lester and Sue Smith Breast Center, Baylor College of Medicine, One Baylor Plaza, Houston, Texas, USA.; 7 CAS Key Laboratory of Tissue Microenvironment and Tumor, Shanghai Institute of Nutrition and Health, Chinese Academy of Sciences, Shanghai, China.; 8 Institute of Biosciences and Technology, Texas A&M Health Science Center, Houston, Texas, USA.; 9 Department of Oncology and Metabolism, The University of Sheffield, Sheffield, United Kingdom.

**Keywords:** Bone Biology, Cell Biology, Bone disease, Breast cancer, Signal transduction

## Abstract

Therapeutics targeting osteoclasts are commonly used treatments for bone metastasis; however, whether and how osteoclasts regulate premetastatic niche and bone tropism are largely unknown. In this study, we report that osteoclast precursors (OPs) can function as a premetastatic niche component that facilitates breast cancer (BCa) bone metastasis at early stages. At the molecular level, unbiased GPCR ligand/agonist screening in BCa cells suggested that R-spondin 2 (RSPO2) and RANKL, through interaction with their receptor LGR4, promoted osteoclastic premetastatic niche formation and enhanced BCa bone metastasis. This was achieved by RSPO2/RANKL-LGR4 signal modulating the WNT inhibitor DKK1 through G**α**_q_ and **β**-catenin signaling. DKK1 directly facilitated OP recruitment through suppression of its receptor LDL receptor–related protein 5 (LRP5) but not LRP6, upregulating *Rnasek* expression via inhibition of canonical WNT signaling. In clinical samples, RSPO2, LGR4, and DKK1 expression showed a positive correlation with BCa bone metastasis. Furthermore, soluble LGR4 extracellular domain (ECD) protein, acting as a decoy receptor for RSPO2 and RANKL, significantly alleviated bone metastasis and osteolytic lesions in a mouse bone metastasis model. These findings provide unique insights into the functional role of OPs as key components of the premetastatic niche for BCa bone metastasis and identify RSPO2/RANKL-LGR4 signaling as a promising target for inhibiting BCa bone metastasis.

## Graphical abstract

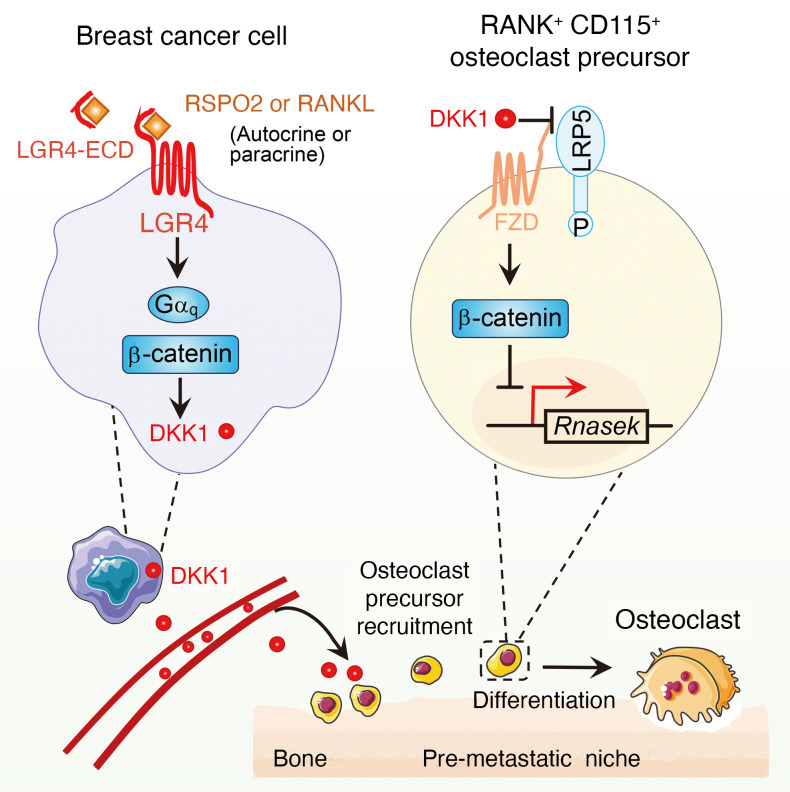

## Introduction

Accumulating evidence demonstrates that target organs of cancer metastasis are not just the passive receivers of disseminated tumor cells, but instead are selectively and positively groomed by the primary tumor before cancer cells have spread beyond the initial site (1). The microenvironment in target organs created by a primary tumor for subsequent metastases is called the premetastatic niche. Several cell types have been verified for their contribution to premetastatic niche formation. Besides vascular cells and cancer-associated fibroblasts, most premetastatic niche component cells are immune cells (2). Although a number of cellular and molecular components have been identified as being involved in premetastatic niche formation in different cancer types and different target organs, the key cells of the premetastatic niche for bone metastasis are largely unknown.

Bone is the metastatic site most commonly targeted by breast cancer (BCa), and bone metastases occur in 75% of metastatic BCa patients (3, 4). In osteolytic BCa bone metastasis, the role of osteoclasts and induction of osteoclastogenesis has been described (5, 6). Development of bone metastases occurs via a “vicious cycle” in which osteoclast-modulated bone resorption releases growth factors stored in bone tissue. These growth factors in turn stimulate metastatic cancer cell survival and growth (5). During osteoclast-modulated bone resorption, it has been well established that RANKL, the ligand for the receptor RANK, and macrophage colony-stimulating factor 1 (CSF-1, also known as M-CSF), the ligand for the receptor CSF1R (CD115), are the 2 critical growth factors for osteoclast differentiation and function (5). Therefore, osteoclast-targeting therapy is extensively used for osteolytic bone metastasis in the clinic, including denosumab, a RANKL antibody that blocks osteoclast differentiation and resorption, and bisphosphonate, a potent small-molecule inhibitor of osteoclast-mediated bone resorption (7, 8). However, the factors involved in initial bone metastasis formation, and the mechanism by which the primary tumor grooms the premetastatic niche, remain unclear. It has been reported that estrogen receptor–negative BCa cells secreted lysyl oxidase, which disrupts normal bone homeostasis and promotes the formation of focal premetastatic lesions by inducing osteoclast differentiation and inhibiting osteoblast proliferation (9). All of these suggest a potential role for osteoclasts in lesion formation. However, whether and how osteoclast precursors (OPs) can be recruited to act as a key premetastatic niche component to regulate BCa bone metastasis at early stages are unknown.

In addition to its role in regulating osteoclast differentiation and function, RANKL-RANK signaling plays an important role in cancer cell bone metastasis (10, 11). RANKL secreted by BCa cells promotes their own growth and eventual metastasis to the bone, where BCa-secreted RANKL also induces osteoclast activation and bone resorption, driving the vicious cycle described above (12, 13). However, whether RANKL signaling plays a role in preparing bone tissue to receive metastases is still unclear.

In this study, R-spondin 2 (RSPO2) and RANKL were identified as critical factors secreted by BCa cells for recruiting OPs and promoting osteoclastic premetastatic niche formation. RSPO2 and RANKL interacted with their receptor LGR4 to modulate the expression of DKK1 through Gα_q_ and WNT/β-catenin signaling in BCa cells. DKK1 overexpression in BCa cells rescued the inhibitory effect of knocking down *LGR4* on osteoclastic premetastatic niche formation. In OPs, DKK1 functionally interacted with LDL receptor–related protein 5 (LRP5) but not LRP6 and regulated the expression of *Rnasek* via canonical WNT signaling for OP recruitment. Moreover, a positive correlation between LGR4 and DKK1, RSPO2 and DKK1, and RANKL and DKK1 expression was found in BCa bone metastasis patient samples. Finally, targeting LGR4 significantly alleviated bone metastasis burden, and ameliorated osteolytic bone lesions in a mouse bone metastasis model.

## Results

### RSPO2 and RANKL promote osteoclastic premetastatic niche formation and facilitate bone metastasis.

To initially evaluate whether factors secreted by BCa cells prepare bone tissues for metastatic colonization, we collected the conditioned medium of MDA-MB-231 (MDA231) BCa cells, and injected it i.p. into mice every day for 2, 3, or 4 weeks (Supplemental Figure 1A; supplemental material available online with this article; https://doi.org/10.1172/JCI144579DS1). The results showed that the number of cells positive for both RANK and CD115 (OP markers), and osteoclast number and activity (determined as osteoclast surface area and eroded surface), were increased in a time-dependent manner (Supplemental Figure 1, B and C). These data suggested that the conditioned medium of BCa cells could recruit OPs and enhance osteoclast number and activity in tumor-free mice. Because there was no significant difference between 3 weeks and 4 weeks, we chose the 3-week time point for subsequent studies.

Our data showed that i.p. injection of conditioned medium significantly stimulated OPs in trabecular rich regions in tibiae and spines of conditioned medium–stimulated mice, compared with control medium–stimulated mice (Supplemental Figure 1, D and E), while negligible numbers of OPs were found in pelvic bones and the skull (Supplemental Figure 1, F and G). Next, we stained osteoclasts by tartrate-resistant acid phosphatase (TRAP) staining in these bones. Our data showed that osteoclast number and activity were increased in both tibiae and spine bones, but not in pelvic or skull bones, in comparison with control medium (Supplemental Figure 1, D–G). To determine whether the osteoclastic premetastatic niche facilitated tumor cell bone metastasis, we used in vitro and in vivo experiments. The in vitro assay showed that the BCa cells could be attracted by OPs in a concentration-dependent manner (Supplemental Figure 2A). Consistently, 3 weeks of pretreatment with conditioned medium, prior to intracardiac (i.c.) injection of luciferase-expressing (LUC-expressing) MDA231 bone-tropic subline SCP46 cells, accelerated the onset of bone metastasis, promoted metastatic burden and osteolytic bone lesions, and increased OP and osteoclast number in tibiae and spines in comparison with control medium–injected mice (Supplemental Figure 2, B–G). However, the pelvis and skull had fewer OPs and few cancer cells (Supplemental Figure 2, H–K). Moreover, the number of cancer cells (stained positive for human vimentin) was significantly associated with the number of OPs/osteoclasts (stained positive for RANK) in the bone (Supplemental Figure 2L). All our results indicated that the osteoclastic premetastatic niche facilitates bone metastasis.

**Figure 1:**
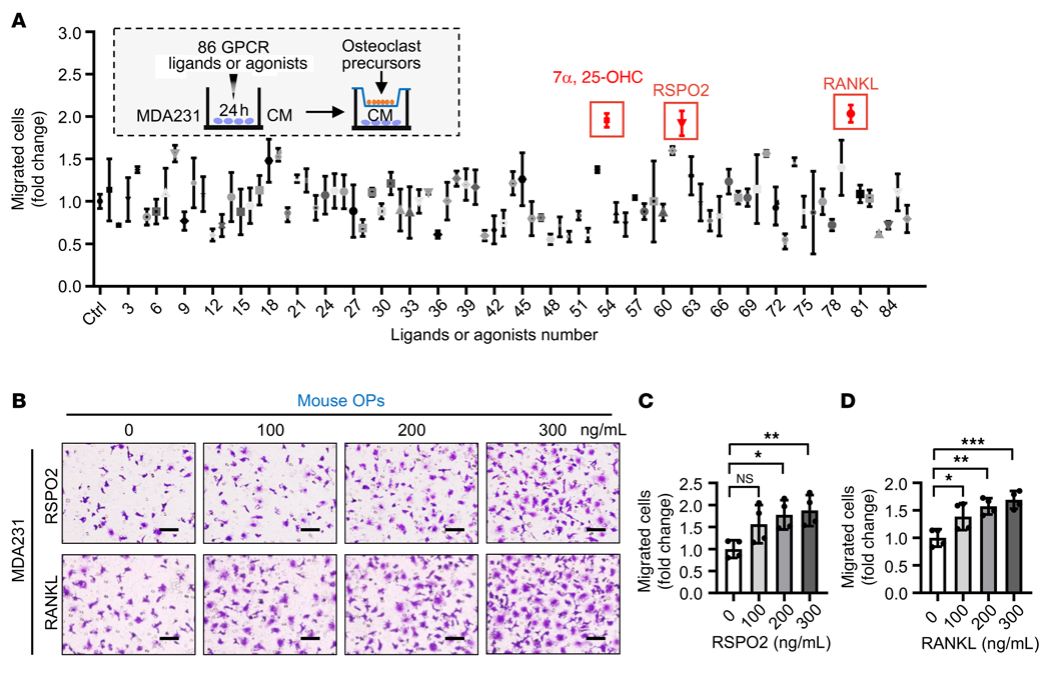
RSPO2 or RANKL promoted OP recruitment by BCa cells. (**A**) Experimental design to screen which GPCRs could recruit OP cells. MDA231 cells were stimulated by 86 GPCR ligands or agonists for 24 hours. Transwell inserts seeded with OP cells (RAW264.7 cells) were then placed in each well. After 16 hours, the migrated RAW264.7 cells were fixed, stained, and counted. Three GPCR ligands (7α,25-OHC, RSPO2, and RANKL) significantly enhanced OP migration using the Transwell migration assay (greater than or equal to 2-fold compared with control [Ctrl]). Every GPCR ligand or agonist was tested in 2 technical replicates. CM, conditioned medium. (**B**) Representative images of primary cultured OPs subjected to the Transwell migration assay (top chamber). (**C** and **D**) MDA231 cells generated conditioned medium following treatment by RSPO2 or RANKL for 24 hours (bottom chamber), with quantification of the number of migrated cells. Data indicate the mean ± SD. *P < 0.05, **P < 0.01, ***P < 0.01 by 1-way ANOVA followed by Dunnett’s post hoc test. *n* = 3 biological replicates. Scale bars: 20 μm.

To identify potential upstream regulators involved in osteoclastic premetastatic niche formation, we performed an unbiased GPCR ligand/agonist screen using an in vitro OP recruitment model (Figure 1A). Specifically, MDA231 cells were stimulated with 86 GPCR ligands/agonists for 24 hours, which correspond to 85 GPCR receptors (Supplemental Table 1), and the conditioned medium was collected for testing the recruitment of OP RAW264.7 cells. We found that conditioned medium from 3 treatment groups (RSPO2, ligand for LGR4/5/6; RANKL, ligand for LGR4 and RANK; 7α,25-OHC, ligand for GPR183) induced OP recruitment greater than or equal to 2-fold higher than control. However, 7α,25-OHC alone but not RSPO2 and RANKL could directly recruit OPs independent of BCa cell stimulation (Supplemental Figure 3A), suggesting that only RSPO2 and RANKL mimicked premetastatic niche formation in vitro. Furthermore, conditioned medium from 4 different BCa cell lines (MDA231, MCF-7, AT3, and 4T1) stimulated with RSPO2 and RANKL induced recruitment of primary cultured OPs (Figure 1, B–D, and Supplemental Figure 3B). Moreover, only RSPO2 and RANKL had this effect among LGR ligands including RSPO1–4, NORRIN, and RANKL (Supplemental Figure 3C).

**Figure 2:**
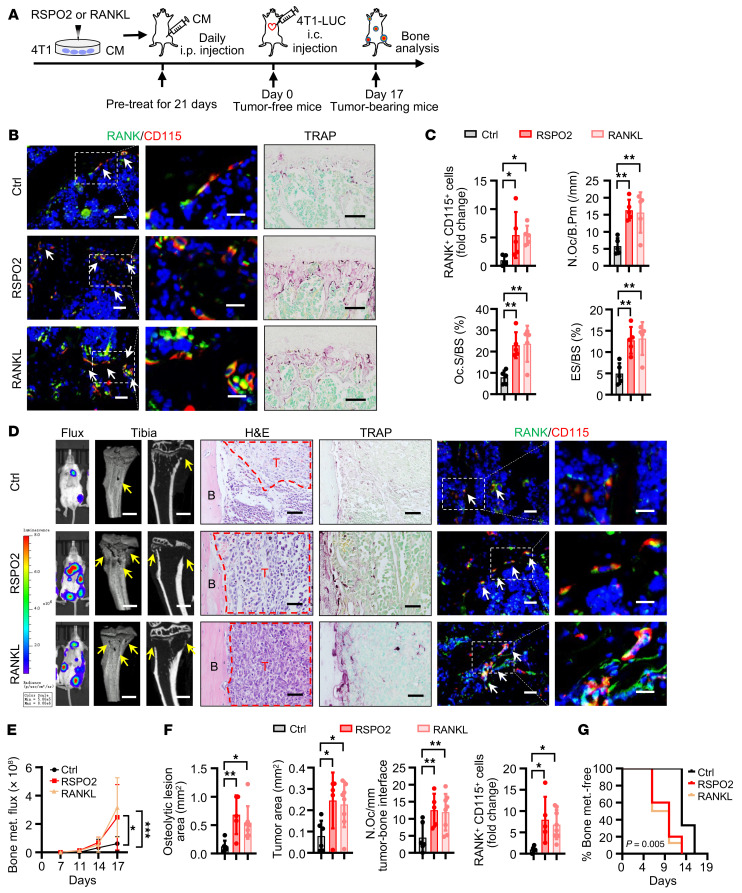
RSPO2 or RANKL promoted osteoclastic premetastatic niche formation by BCa cells. (**A**) Experimental design of the in vivo premetastatic niche mouse model. 4T1 cells were treated with RSPO2 or RANKL; then conditioned medium was collected and injected i.p. into BALB/c mice every day for 21 days of pretreatment. Then mice were either euthanized and bones harvested (tumor-free mice), or 4T1-LUC cells were injected i.c. into the mice (day 0). Seventeen days after i.c. injection, the mice (tumor-bearing mice) were sacrificed and bones analyzed. (**B** and **C**) Representative images of immunofluorescence (IF) double staining for RANK (green) and CD115 (red) and TRAP staining in the third lumbar vertebrae (L3 spine) of tumor-free mice (**B**) and quantitative analysis (**C**). White arrows indicate cells double-positive for RANK and CD115. Data indicate the mean ± SD. *P < 0.05, **P < 0.01, 1-way ANOVA followed by Dunnett’s post hoc test. *n* = 5 per group. Scale bars: 10 μm/5 μm (left/right, IF), 50 μm (TRAP). N.Oc/B.Pm, osteoclast number/bone perimeter; Oc.S/BS, osteoclast surface/bone surface; ES/BS, eroded surface/bone surface. (**D**–**G**) Representative images of bioluminescent, radiographic, H&E, TRAP, and IF double staining for RANK (green) and CD115 (red) in the tibiae of tumor-bearing mice (**D**) and quantitative analysis (**E**–**G**). Yellow arrows indicate osteolytic lesions; red dotted lines indicate tumor zone; white arrows indicate cells double-positive for RANK and CD115. Data indicate the mean ± SD. *P < 0.05, **P < 0.01, ***P < 0.001, (**E**) 2-way ANOVA, (**F**) 1-way ANOVA followed by Dunnett’s post hoc test, (**G**) log-rank test. Ctrl, *n* = 6; RSPO2, *n* = 5; RANKL, *n* = 8. Scale bars: 1 mm (micro-CT), 100 μm (TRAP), 25 μm (H&E), 10 μm/5 μm (left/right, IF). T, tumor; B, bone.

It is well established that RANKL is a key cytokine affecting the immune system, including T cells, B cells, and macrophages (14–16), and it has been reported that RSPO signaling promotes macrophage polarization in the tumor microenvironment (17). To systematically evaluate the role of RSPO2 and RANKL in the premetastatic niche, we used immunocompetent mice with the mouse mammary cancer cell line 4T1 (Figure 2A). After 4T1 cells were stimulated by RSPO2 or RANKL, the conditioned medium was collected and then injected i.p. into mice every day. After 21 days, OP number and osteoclast number and activity were increased in both the group treated with RSPO2-stimulated conditioned medium and the group treated with RANKL-stimulated conditioned medium (tumor-free mice) (Figure 2, B and C). To determine whether the osteoclastic premetastatic niche facilitated tumor cell bone metastasis, we injected LUC-expressing 4T1 cells i.c. into all groups of mice at day 21. Mice pretreated with RSPO2- or RANKL-stimulated conditioned medium had a dramatic increase in bone metastatic burden as determined by bioluminescent imaging (Figure 2, D and E), aggravated osteolytic bone lesions (Figure 2, D and F), and an earlier onset of bone metastasis (Figure 2G). Consistent with these results, OP number and osteoclast number were increased in the groups pretreated with RSPO2- or RANKL-stimulated conditioned medium (Figure 2, D and F). Furthermore, RSPO2- or RANKL-stimulated conditioned medium had no significant effect on BCa cell metastasis to lung, kidney, and brain (Supplemental Figure 3D). Together, these results demonstrated that RSPO2 and RANKL promoted osteoclastic premetastatic niche formation and ultimately enhanced BCa bone metastasis.

### LGR4 regulates osteoclastic premetastatic niche formation and bone metastasis.

**Figure 3:**
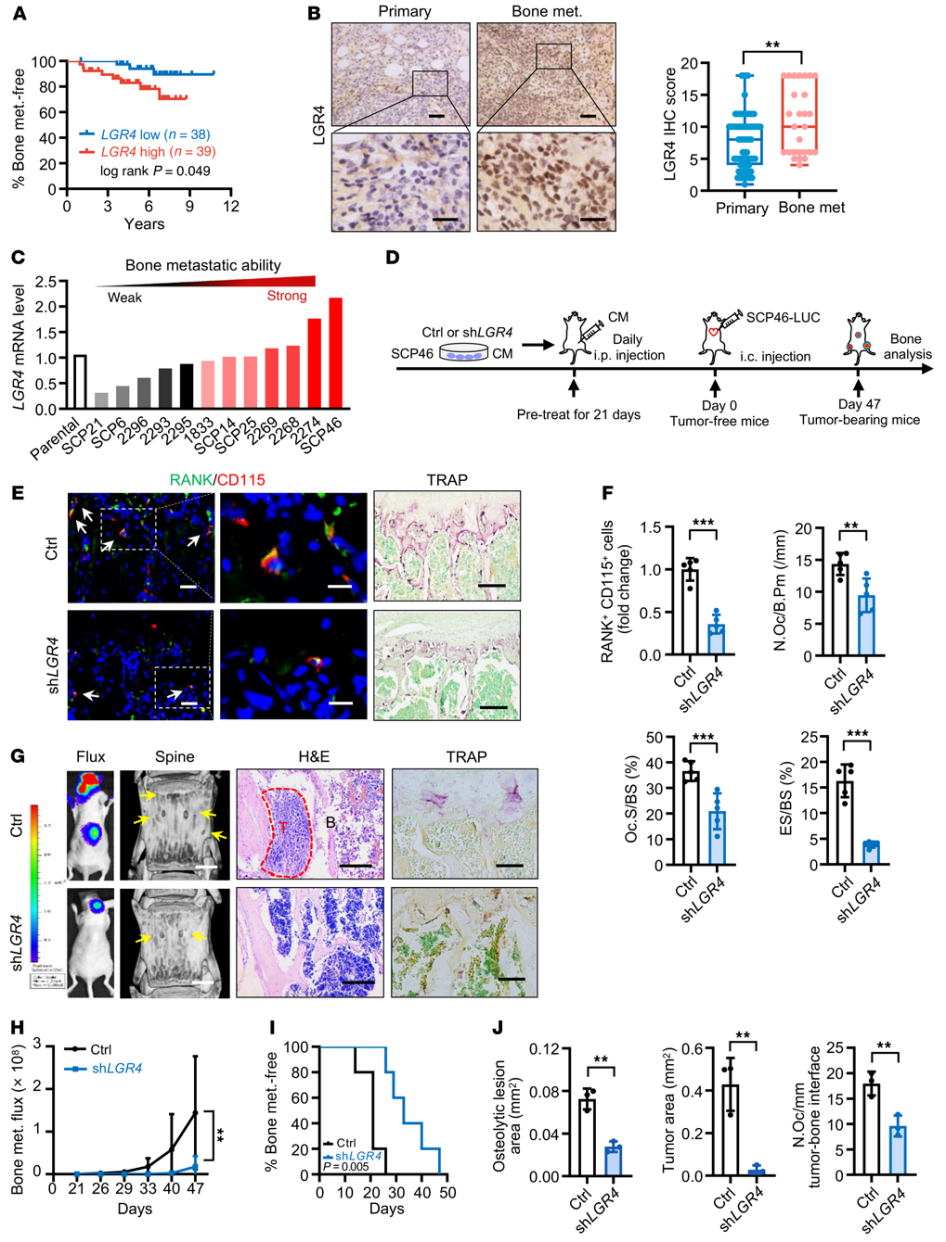
LGR4 contributed to OP recruitment and regulated osteoclastic premetastatic niche formation and bone metastasis. (**A**) Kaplan-Meier analysis of bone metastasis–free survival according to *LGR4* mRNA expression in BCa patients (GEO GSE2603; *LGR4* low, *n* = 38; *LGR4* high, *n* = 39). Log-rank test. (**B**) LGR4 IHC staining in BCa patient samples and quantitative analysis. Data indicate the mean ± SD. **P < 0.01, unpaired 2-tailed Student’s *t* test. Primary, *n* = 73; bone met., *n* = 27. Scale bars: 50 μm/20 μm (top/bottom). (**C**) *LGR4* expression was correlated with bone metastatic capability in MDA231 sublines with distinct bone-metastasis abilities (GEO GSE14244 and GSE16554). (**D**) Experimental design of the in vivo premetastatic niche mouse model. Conditioned medium from *LGR4*-knockdown SCP46 cells was collected and injected i.p. into nude mice every day for 21 days of pretreatment (tumor-free mice); SCP46-LUC cells were injected i.c. into mice (day 0). Forty-seven days after i.c. injection, bones of mice (tumor-bearing mice) were analyzed. (**E** and **F**) Representative images of IF double staining for RANK (green) and CD115 (red) and TRAP staining in the L3 spines of tumor-free mice (**E**), and quantitative analysis (**F**). White arrows, double-positive for RANK and CD115. Data indicate the mean ± SD. **P < 0.01, ***P < 0.001, unpaired 2-tailed Student’s *t* test, *n* = 5. Scale bars: 10 μm/5 μm (left/right, IF), 50 μm (TRAP). (**G**–**J**) Representative images of bioluminescent, radiographic, H&E, and TRAP staining in the L3 spines of tumor-bearing mice (**G**), and quantitative analysis (**H**–**J**). Yellow arrows, osteolytic lesions; red dotted lines, tumor zone. Data indicate the mean ± SD. **P < 0.01, (**H**) 2-way ANOVA, (**I**) log-rank test, *n* = 5 (**H** and **I**); (**J**) unpaired 2-tailed Student’s *t* test, *n* = 3. Scale bars: 1 mm (micro-CT), 200 μm (H&E), 30 μm (TRAP).

LGR4, LGR5, and LGR6 are receptors for RSPO2 (18), while RANK and LGR4 are receptors for RANKL (19, 20). We next asked which receptor(s) of RSPO2 and RANKL are involved in regulating premetastatic niche formation. We first examined the clinical significance of each receptor (LGR4/5/6) of RSPO2 in human BCa bone metastasis in a previously published data set (21). Because there is no signal of *LGR6* in this data set and the expression of *LGR6* is extremely low in BCa cell lines (22), we only analyzed the relationship of LGR4 and LGR5 to Kaplan-Meier bone metastasis–free survival of BCa patients. Our results indicated that only high expression of *LGR4*, but not *LGR5*, was significantly associated with the incidence of bone metastasis (Figure 3A and Supplemental Figure 4A). Then, we asked which RANKL receptor, LGR4 or RANK, was responsible for OP recruitment. Our data showed that knockdown of *LGR4*, but not *RANK*, in cancer cells dramatically decreased the RANKL-induced recruitment of primary cultured OPs (Supplemental Figure 4, B and C). Therefore, we focused on the overlapping receptor LGR4 for the following study.

Our IHC staining results confirmed that the expression level of LGR4 was significantly higher in human bone metastatic tumor samples (*n* = 27) compared with primary breast tumor samples (*n* = 73; Figure 3B). Furthermore, the expression level of *LGR4* was correlated with bone metastatic capability in a series of extensively used MDA231 human BCa bone-tropic sublines (13 sublines) with distinct bone-metastasis abilities (refs. 23, 24; Figure 3C; and Supplemental Table 2). Silencing the expression of *LGR4* in two BCa cell lines (MDA231 and BT549), or using *Lgr4*-heterozygous (*Lgr4^+/−^*) primary cultured mouse PyMT BCa cells, resulted in conditioned medium with strikingly reduced OP recruitment (Supplemental Figure 4, D and E). Moreover, re-expression of *LGR4* in the same cancer cells (MDA231 and BT549) restored OP recruitment (Supplemental Figure 4D). Furthermore, conditioned medium from MCF-7 cells overexpressing LGR4 remarkably enhanced OP recruitment (Supplemental Figure 4F).

We next collected the conditioned medium of *LGR4*-knockdown SCP46 cells, a highly efficient bone tropism cell line with the highest *LGR4* expression level (Figure 3C), and injected it i.p. into the in vivo premetastatic niche mouse model every day for 3 weeks (Figure 3D). After 21 days, the bones of the tumor-free mice were sectioned and analyzed. Our data showed that OP number and osteoclast number and activity were decreased in the *LGR4*-knockdown group (Figure 3, E and F). To examine whether the *LGR4* knockdown–induced change to the osteoclastic premetastatic niche would result in inhibition of BCa cell bone metastasis, we performed i.c. injection of LUC-expressing SCP46 cells into all groups of mice at day 21. As expected, *LGR4* knockdown strikingly inhibited metastatic burden in the spines, protected against osteolytic bone lesions, and delayed the onset of bone metastasis (Figure 3, G–J). These results demonstrated that the RSPO2 and RANKL receptor LGR4 is a key regulator of formation of the osteoclastic premetastatic niche and subsequent bone metastasis.

### DKK1 is the downstream target of LGR4 that regulates osteoclastic premetastatic niche formation and bone metastasis.

**Figure 4:**
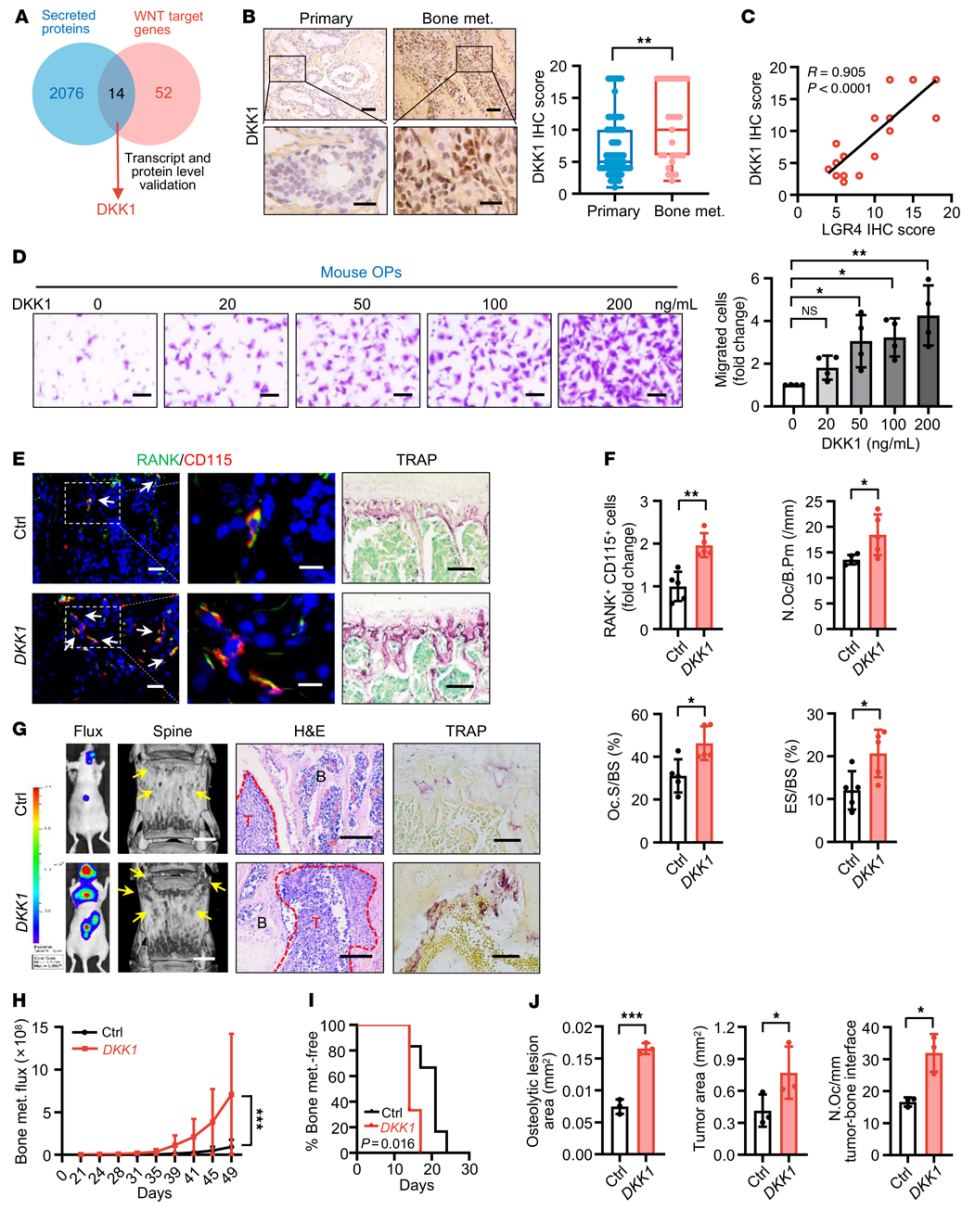
DKK1 was identified as the key factor for osteoclastic premetastatic niche formation and bone metastasis. (**A**) Fourteen targets were identified as secreted proteins regulated by WNT signaling. (**B**) DKK1 IHC staining in BCa patients and quantitative analysis. Data indicate the mean ± SD. **P < 0.01, unpaired 2-tailed Student’s *t* test. Primary, *n* = 73; bone met., *n* = 27. Scale bars: 50 μm/20 μm (top/bottom). (**C**) Correlation of LGR4 expression with DKK1 expression in BCa bone metastasis tumors. Pearson’s correlation analysis. (**D**) Primary cultured OPs (top chamber) were subjected to migration assay with DKK1 (bottom chamber) and quantified. Data indicate the mean ± SD. *P < 0.05, **P < 0.01, 1-way ANOVA with Dunnett’s post hoc test. *n* = 4 biological replicates. Scale bars: 20 μm. (**E **and **F**) Conditioned medium from DKK1-overexpressing SCP46 cells was injected i.p. into BALB/c nude mice every day for 21 days (tumor-free mice). IF double staining for RANK (green) and CD115 (red), TRAP staining in L3 spines of mice (**E**), and quantitative analysis (**F**). White arrows, cells double-positive for RANK and CD115. Data indicate the mean ± SD. *P < 0.05, **P < 0.01, unpaired 2-tailed Student’s *t* test, *n* = 5. Scale bars: 10 μm/5 μm (left/right, IF), 50 μm (TRAP). (**G**–**J**) SCP46-LUC cells were injected i.c. into tumor-free mice (day 0); 49 days later, bones of tumor-bearing mice were analyzed. Representative images of bioluminescent, radiographic, H&E, and TRAP staining in L3 spines of mice (**G**), and quantitative analysis (**H**–**J**). Yellow arrows, osteolytic lesions; red dotted lines, tumor zone. Data indicate the mean ± SD. *P < 0.05, ***P < 0.001, (**H**) 2-way ANOVA, (**I**) log-rank test, *n* = 6 (**H** and **I**); (**J**) unpaired 2-tailed Student’s *t* test, *n* = 3. Scale bars: 1 mm (micro-CT), 200 μm (H&E), 30 μm (TRAP).

Next, we asked which secreted factor modulated by RSPO2/RANKL-LGR4 signaling regulates osteoclastic premetastatic niche formation. We identified 2090 human secreted proteins from the UniProt database by bioinformatic analysis (Supplemental Table 3 and Supplemental Methods). It is well established that RSPO-LGR4 signals through the WNT pathway, with 66 confirmed WNT target genes identified in a colon cancer study (25). Overlapping these 2 sets of genes, we found 14 genes that encode secreted proteins regulated by WNT/β-catenin signaling (Figure 4A). We next compared the expression of these 14 genes in *LGR4*-knockdown SCP46 cells and found that the *DKK1* transcript level was one of the most highly changed (Supplemental Table 4). Proteomic profiling of proteins in conditioned medium from *LGR4* WT and knockout MDA-MB-468 (MDA468) BCa cells showed that, of the 14 proteins, DKK1 had the highest change in level (Supplemental Table 5). Regulation of *DKK1* expression was further confirmed in *LGR4*-silenced MDA231, MDA468, BT549, and SCP46 cells using reverse transcriptase PCR (RT-PCR) or quantitative RT-PCR (Supplemental Figure 5, A and B). Furthermore, DKK1 expression was significantly higher in human bone metastatic tumor samples (*n* = 27) compared with primary breast tumor samples (*n* = 73) by IHC staining (Figure 4B). In contrast, there was no significant difference in the expression of DKK1 in human lymph node metastatic tumor samples (*n* = 50) compared with primary BCa samples (*n* = 50; Supplemental Figure 5C). We also found a positive correlation between LGR4 (Figure 3B) and DKK1 (Figure 4B) expression in our bone metastatic samples (*n* = 27, *P* < 0.0001; Figure 4C). A similar correlation was present between the mRNA levels of *DKK1* and *LGR4* in the previously mentioned 13 MDA231 cell sublines (Supplemental Figure 5D and Supplemental Table 2), and 8 distinct human BCa cell lines (Supplemental Figure 5E and Supplemental Table 6).

Our data also showed that DKK1 treatment directly led to OP recruitment in a dose-dependent manner in both primary cultured OPs (Figure 4D) and RAW264.7 cells (Supplemental Figure 5, F and G), while DKK1 had only a mild effect on OP differentiation into mature osteoclasts at high concentration (Supplemental Figure 5, H and I). In the in vivo premetastatic niche mouse model, conditioned medium of *DKK1*-overexpressing SCP46 cancer cells remarkably induced OP number and increased osteoclast number and activity compared with vector control cancer cell conditioned medium in tumor-free mice at day 21 (Figure 4, E and F). In the BCa cell i.c. injection metastasis model, pretreatment with *DKK1* overexpression conditioned medium significantly promoted bone metastasis burden, increased osteolytic bone lesions, and accelerated bone metastasis (Figure 4, G–J). To further verify the contribution of DKK1 to the enhanced osteoclastic premetastatic niche and BCa cell bone metastasis, we collected the conditioned medium of *DKK1*-overexpressing MDA231 subline SCP6, a poorly efficient bone tropism cell line with lower *LGR4* and *DKK1* expression levels (Figure 3C and Supplemental Table 2). The conditioned medium was then injected i.p. into the in vivo premetastatic niche mouse model every day for 3 weeks, followed by i.c. injection of LUC-expressing SCP6 cells. As expected, *DKK1* overexpression conditioned medium strikingly promoted bone metastatic burden caused by poorly efficient bone-tropic SCP6-LUC cells, increased osteolytic bone lesions, accelerated the onset of bone metastasis, and increased OP and osteoclast numbers (Supplemental Figure 5, J–M), indicating that conditioned medium pretreatment of the mice changed the behavior of the LUC-SPC6 cells, and DKK1 was a critical mediator of osteoclastic premetastatic niche formation and bone metastasis.

### RSPO2/RANKL-LGR4 regulates osteoclastic premetastatic niche formation and bone metastasis by modulating DKK1 expression.

**Figure 5:**
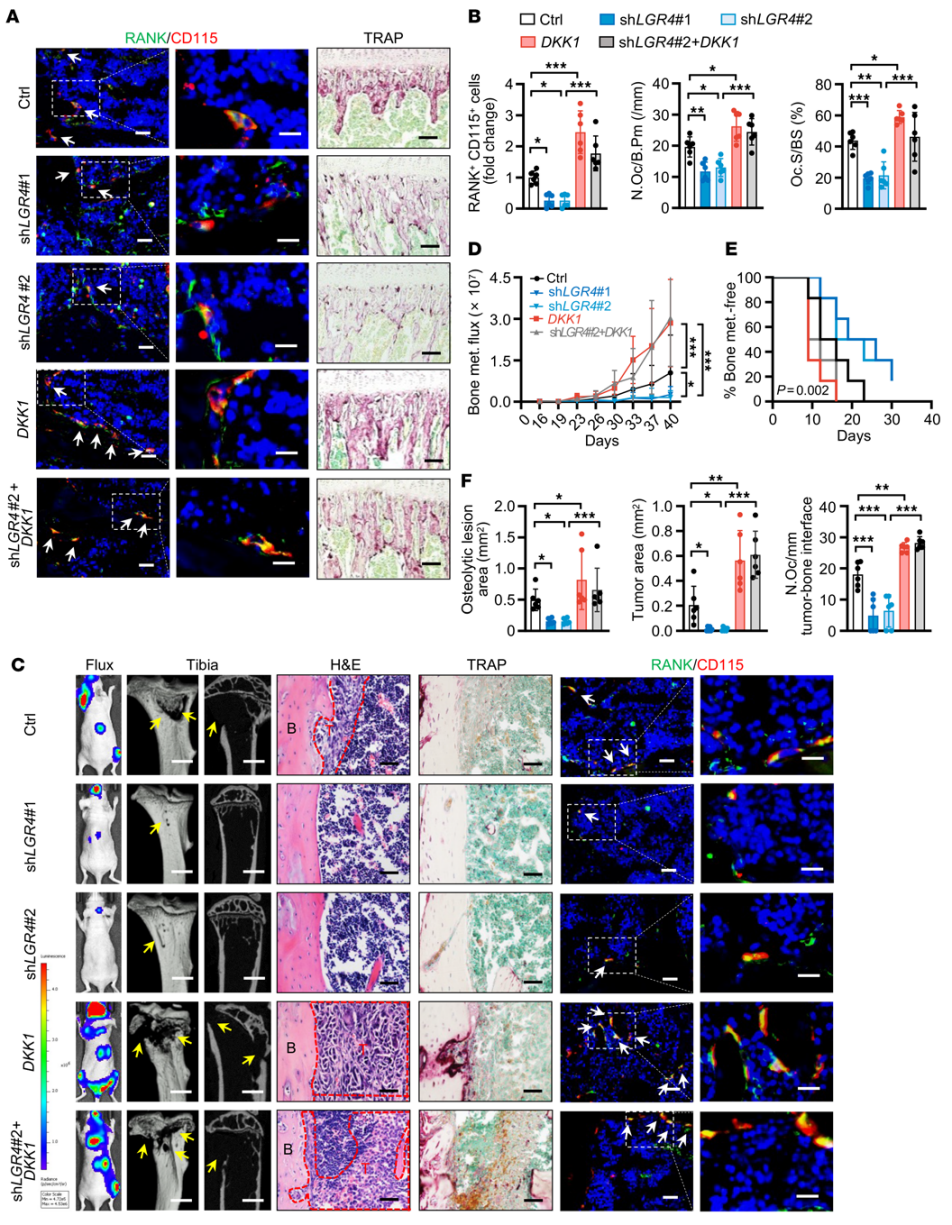
DKK1 rescued the loss of effect on OP recruitment and osteoclastic premetastatic niche formation following *LGR4* knockdown. (**A** and **B**) Knockdown of *LGR4*, transfection of *DKK1* expression plasmid, or exogenous expression of *DKK1* with *LGR4* knockdown was performed in SCP46 cells, the conditioned medium was collected and injected i.p. into nude mice every day for 21 days of pretreatment, and tumor-free mice were euthanized at this time point. Representative images of IF double staining for RANK (green) and CD115 (red) and TRAP staining in the tibiae of mice (**A**), and quantitative analysis (**B**). White arrows indicate cells double-positive for RANK and CD115. Data indicate the mean ± SD. *P < 0.05, **P < 0.01, ***P < 0.001, 1-way ANOVA with Tukey’s post hoc test. *n* = 6 per group. Scale bars: 10 μm/5 μm (left/right, IF), 50 μm (TRAP). (**C**–**F**) SCP46-LUC cells were injected i.c. into tumor-free mice (tumor-bearing mice); 40 days later, the mice were sacrificed and bone analysis was performed. Representative images of bioluminescent, radiographic, H&E, TRAP, and IF double staining for RANK (green) and CD115 (red) in the tibiae of mice (**C**), and quantitative analysis (**D**–**F**). Yellow arrows indicate osteolytic lesions; red dotted lines indicate tumor zones; white arrows indicate cells double-positive for RANK and CD115. Data indicate the mean ± SD. *P < 0.05, **P < 0.01, ***P < 0.001, (**D**) 2-way ANOVA, (**E**) log-rank test, (**F**) 1-way ANOVA with Tukey’s post hoc test. *n* = 6 per group. Scale bars: 1 mm (micro-CT), 50 μm (H&E and TRAP), 10 μm/5 μm (left/right, IF).

To further explore whether LGR4 regulated osteoclastic premetastatic niche formation and bone metastasis by modulating DKK1 expression, we used a “rescue” strategy in vitro and in vivo. Our data showed that overexpression of *DKK1* in *LGR4*-silenced cancer cells successfully rescued the reduced OP recruitment (Supplemental Figure 6A). Consistent with this, knocking down *DKK1* inhibited the ability of LGR4-overexpressing cancer cells to attract OPs (Supplemental Figure 6B). In the premetastatic niche in vivo model, overexpression of *DKK1* in human SCP46 cancer cells restored the serum level of human DKK1 protein inhibited by *LGR4* knockdown (Supplemental Figure 6, C and D) and reversed the effects of *LGR4* silencing on OP number and osteoclast number and activity (Figure 5, A and B, and Supplemental Figure 6E). Consequently, conditioned medium from cells overexpressing *DKK1* reversed the *LGR4* knockdown–induced decline in bone metastasis burden, osteolytic bone lesions, and the onset of bone metastasis, as well as numbers of OPs and osteoclasts, following i.c. injection of BCa cells (Figure 5, C–F, and Supplemental Figure 6F).

**Figure 6:**
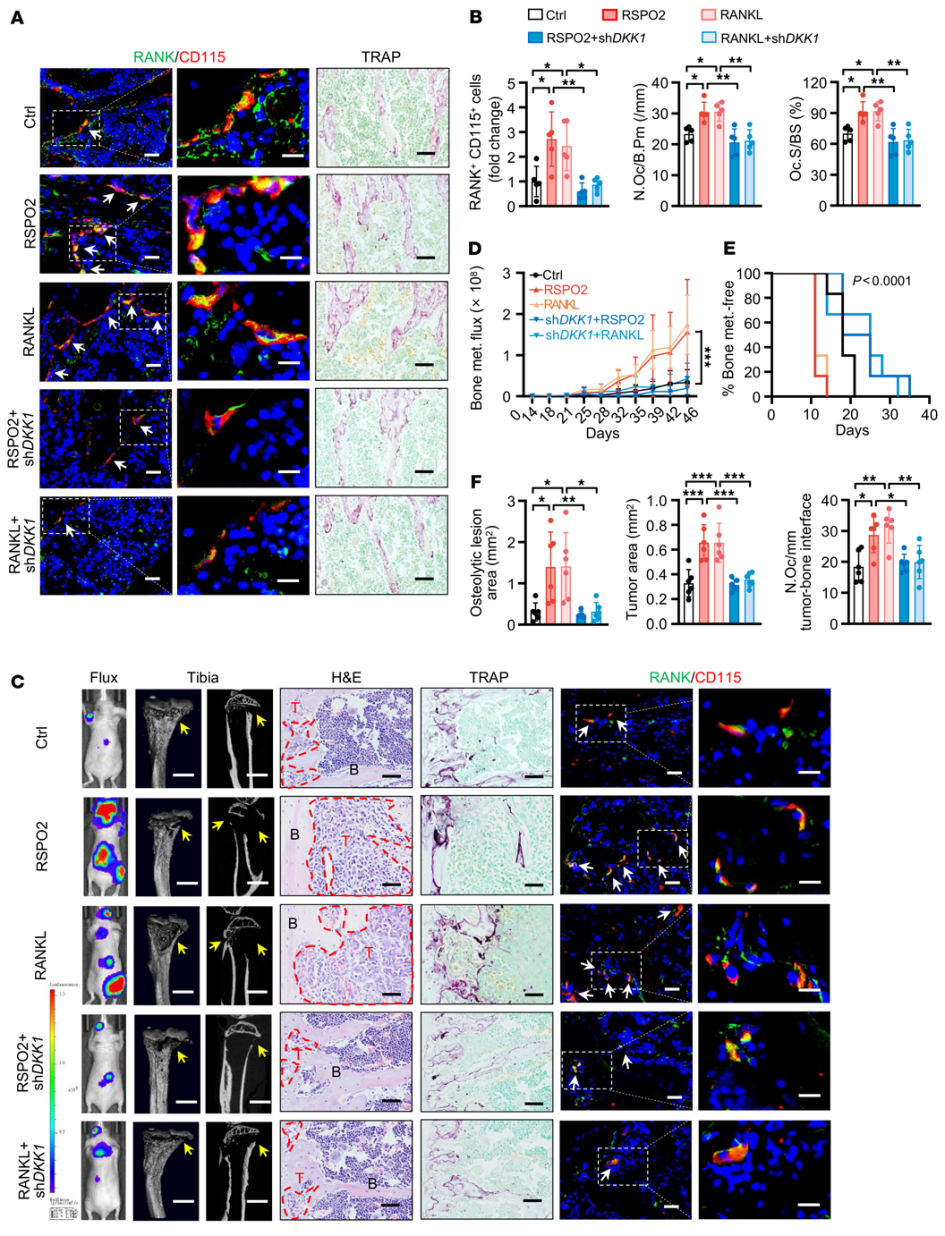
RSPO2 or RANKL regulated osteoclastic premetastatic niche formation and bone metastasis via DKK1 signaling. (**A** and **B**) Conditioned medium (Ctrl, RSPO2, RANKL, RSPO2+sh*DKK1*, RANKL+sh*DKK1*) from SCP46 cells was injected i.p. into nude mice for 21 days (tumor-free mice). Representative images of IF double staining for RANK (green) and CD115 (red) and TRAP staining in the tibiae of mice (**A**), and quantitative analysis (**B**). White arrows indicate cells double-positive for RANK and CD115. Data indicate the mean ± SD. *P < 0.05, **P < 0.01, 1-way ANOVA with Tukey’s post hoc test. *n* = 5 per group. Scale bars: 10 μm/5 μm (left/right, IF), 50 μm (TRAP). (**C**–**F**) Mice of indicated experimental groups were analyzed 46 days after i.c. SCP46-LUC cell injection (tumor-bearing mice). Representative images of bioluminescent, radiographic, H&E, TRAP, and IF double staining for RANK (green) and CD115 (red) in the tibiae of mice (**C**), and quantitative analysis (**D**–**F**). Yellow arrows indicate osteolytic lesion areas; red dotted lines indicate tumor zone; white arrows indicate cells double-positive for RANK and CD115. Data indicate the mean ± SD. *P < 0.05, **P < 0.01, ***P < 0.001, (**D**) 2-way ANOVA, (**E**) log-rank test, (**F**) 1-way ANOVA with Tukey’s post hoc test. *n* = 6 per group. Scale bars: 1 mm (micro-CT), 25 μm (H&E), 50 μm (TRAP), 10 μm/5 μm (left/right, IF).

Next, we examined whether RSPO2/RANKL regulated osteoclastic premetastatic niche formation and bone metastasis via DKK1 signaling. We collected the conditioned medium from SCP46 cancer cells stimulated by RSPO2 or RANKL with or without *DKK1* knockdown and then injected the conditioned medium i.p. into mice every day for 21 days. Our data showed that knockdown of *DKK1* in cancer cells successfully reduced the RSPO2- or RANKL-induced OP recruitment and osteoclast number and activity in tibiae and spines (Figure 6, A and B, and Supplemental Figure 6, G–I). After i.c. injection of BCa cells, the group receiving conditioned medium from *DKK1*-knockdown cells had decreased RSPO2/RANKL-induced bone metastasis burden and osteolytic bone lesions, delayed onset of bone metastasis, and reduced numbers of OPs and osteoclasts (Figure 6, C–F, and Supplemental Figure 6J). Together, our results demonstrated that RSPO2/RANKL-LGR4 regulates DKK1 expression to promote osteoclastic premetastatic niche formation and bone metastasis.

### RSPO2/RANKL-LGR4 regulates DKK1 expression through Gα_q_ and β-catenin signaling in BCa cells.

**Figure 7:**
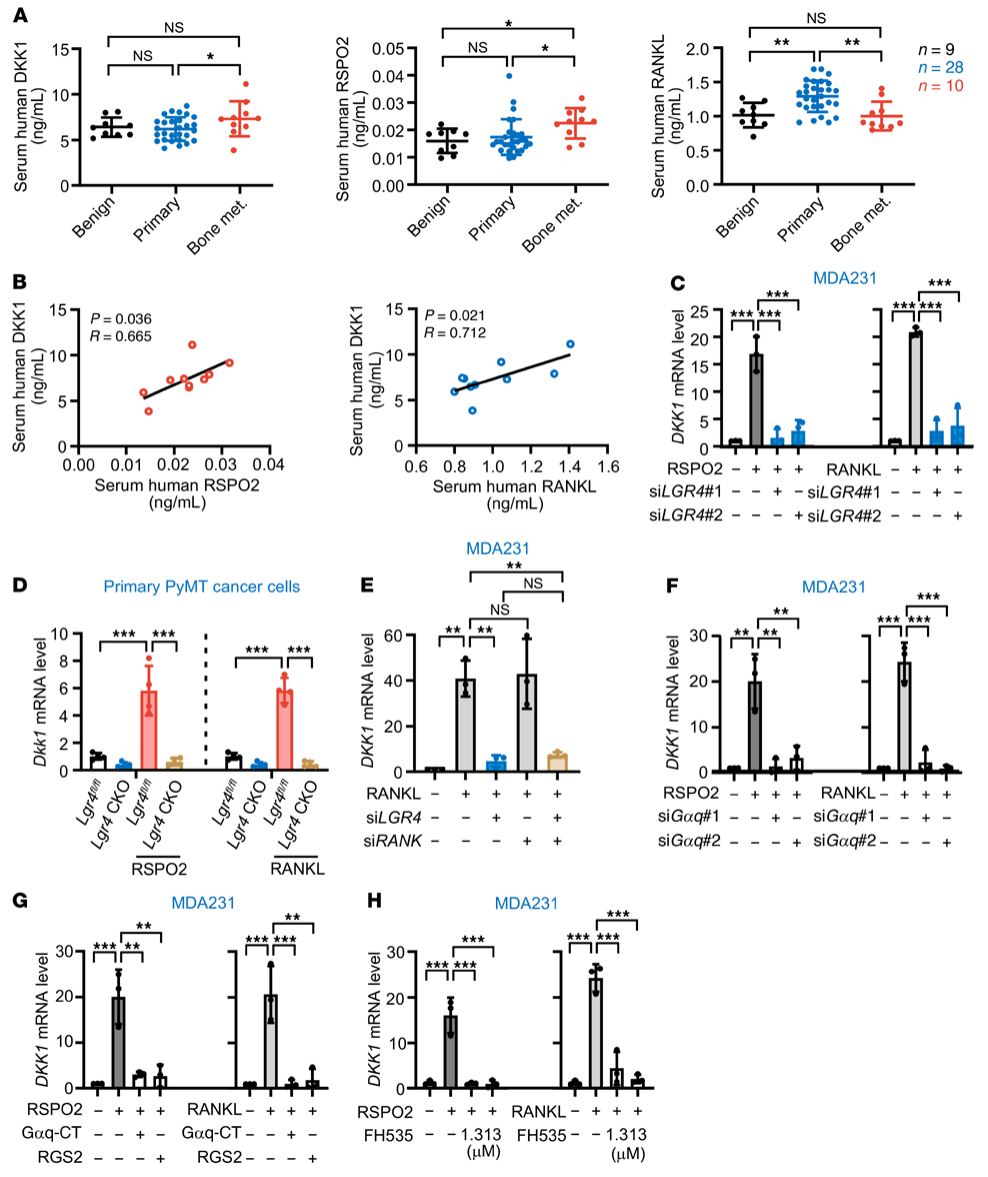
RSPO2/RANKL-LGR4 signaling induced DKK1 expression though Gα_q_ and β-catenin signaling pathway. (**A**) Serum RSPO2/RANKL/DKK1 levels in BCa patients. Data indicate the mean ± SD. *P < 0.05, **P < 0.01, Kruskal-Wallis test. Benign, *n* = 9; primary, *n* = 28; bone metastasis, *n* = 10. (**B**) Serum RSPO2/DKK1 and RANKL/DKK1 correlations in BCa patients with bone metastasis. Pearson’s correlation analysis. (**C**) *DKK1* mRNA levels in MDA231 cells stimulated with RSPO2 (left) or RANKL (right) for 6 hours with or without *LGR4* knockdown. Data indicate the mean ± SD. ***P < 0.001, 1-way ANOVA with Tukey’s post hoc test. *n* = 3 biological replicates. (**D**) *Dkk1* mRNA levels in murine primary cultured tumor cells (MMTV-PyMT *Lgr4^fl/fl^* and MMTV-PyMT *MMTV*-Cre *Lgr4^fl/fl^* [CKO]) stimulated with RSPO2 or RANKL for 6 hours. Data indicate the mean ± SD. ***P < 0.001, 1-way ANOVA with Tukey’s post hoc test. *n* = 4 biological replicates. (**E**) *DKK1* mRNA levels in *LGR4*- or *RANK*-knockdown MDA231 cells following treatment with RANKL for 6 hours. Data indicate the mean ± SD. **P < 0.01, 1-way ANOVA with Tukey’s post hoc test. *n* = 3 biological repeats. (**F** and **G**) *DKK1* mRNA levels in MDA231 cells stimulated with RSPO2 or RANKL for 6 hours with or without 2 siRNAs targeting Gα_q_ or transfected with vectors expressing 2 Gα_q_ inhibitors (the C-terminal domain of Gα_q_ [Gαq-CT] or regulator of G protein signaling 2 [RGS2]). Data indicate the mean ± SD. **P < 0.01, ***P < 0.001, 1-way ANOVA with Tukey’s post hoc test. *n* = 3 biological repeats. (**H**) *DKK1* mRNA levels in MDA231 cells stimulated with RSPO2 (left) or RANKL (right) for 6 hours with or without inhibition of β-catenin. Data indicate the mean ± SD. ***P < 0.001, 1-way ANOVA with Tukey’s post hoc test. *n* = 3 biological replicates.

To examine whether RSPO2/RANKL-DKK1 signaling is specific to bone metastasis patients, we next examined the serological RSPO2, RANKL, and DKK1 levels in benign breast lump patients (*n* = 9), primary BCa patients (*n* = 28), and bone metastasis BCa patients (*n* = 10). The results showed that the expression level of DKK1 was significantly higher in bone metastatic BCa patients compared with primary BCa patients, and RSPO2 expression levels were higher in bone metastatic BCa patients compared with both primary BCa and benign lump patients (Figure 7A). However, the RANKL level, while higher in primary BCa patients, was not higher in bone metastatic BCa patients compared with the benign group, which is consistent with previous reports (26, 27). Interestingly, there was a significant positive correlation between DKK1 and RSPO2, as well as between DKK1 and RANKL, in bone metastatic BCa patients (Figure 7B) but not in benign lump and primary BCa patients (Supplemental Figure 7, A and B). Moreover, in 7 different BCa cell lines including basal-like, luminal, and human epidermal growth factor receptor 2 (HER2) subtypes, stimulation with RSPO2 or RANKL induced *DKK1* expression at the mRNA level (Supplemental Figure 7, C and D), and increases in DKK1 protein level were detected in stimulated MDA231 cells and 4T1 cells (Supplemental Figure 7, E and F). *DKK1* upregulation in the HER2^+^ cell lines was much lower than that in the other subtypes, which is consistent with a clinical report that HER2^+^ BCa has lower bone metastasis propensity (3). Furthermore, from the in vivo premetastatic niche mouse model (Figure 2, B and C), we found that the serum DKK1 level was significantly increased in the RSPO2- and RANKL-stimulated group (Supplemental Figure 7G).

We next examined whether RSPO2- or RANKL-induced DKK1 expression was dependent on LGR4 and sought to clarify the signaling pathways downstream of LGR4. *LGR4* knockdown or knockout almost completely blocked RSPO2 or RANKL induction of DKK1 expression in MDA231 cells (Figure 7C) and primary cultured PyMT cells (Figure 7D), whereas RANKL-induced DKK1 expression was independent of its classical receptor RANK (Figure 7E and Supplemental Figure 7H). RSPO-LGR4 potentiates WNT/β-catenin signaling, and RANKL-LGR4 goes through Gα_q_ signaling (20), so we next investigated whether Gα_q_ or β-catenin signaling regulates DKK1 expression. Using 2 siRNAs and 2 inhibitors of Gα_q_, and 2 concentrations of the β-catenin inhibitor FH535, we found that inhibition of either Gα_q_ or β-catenin almost completely suppressed RSPO2- or RANKL-induced *DKK1* expression (Figure 7, F–H), suggesting that Gα_q_ or β-catenin was required for LGR4 regulation of DKK1 expression. Thus, all these data demonstrated that RSPO2/RANKL-LGR4 induces expression of DKK1 via the Gα_q_ and β-catenin signaling pathways.

### DKK1 functionally interacts with LRP5 but not LRP6 and regulates the expression of Rnasek via canonical WNT signaling for OP recruitment.

**Figure 8:**
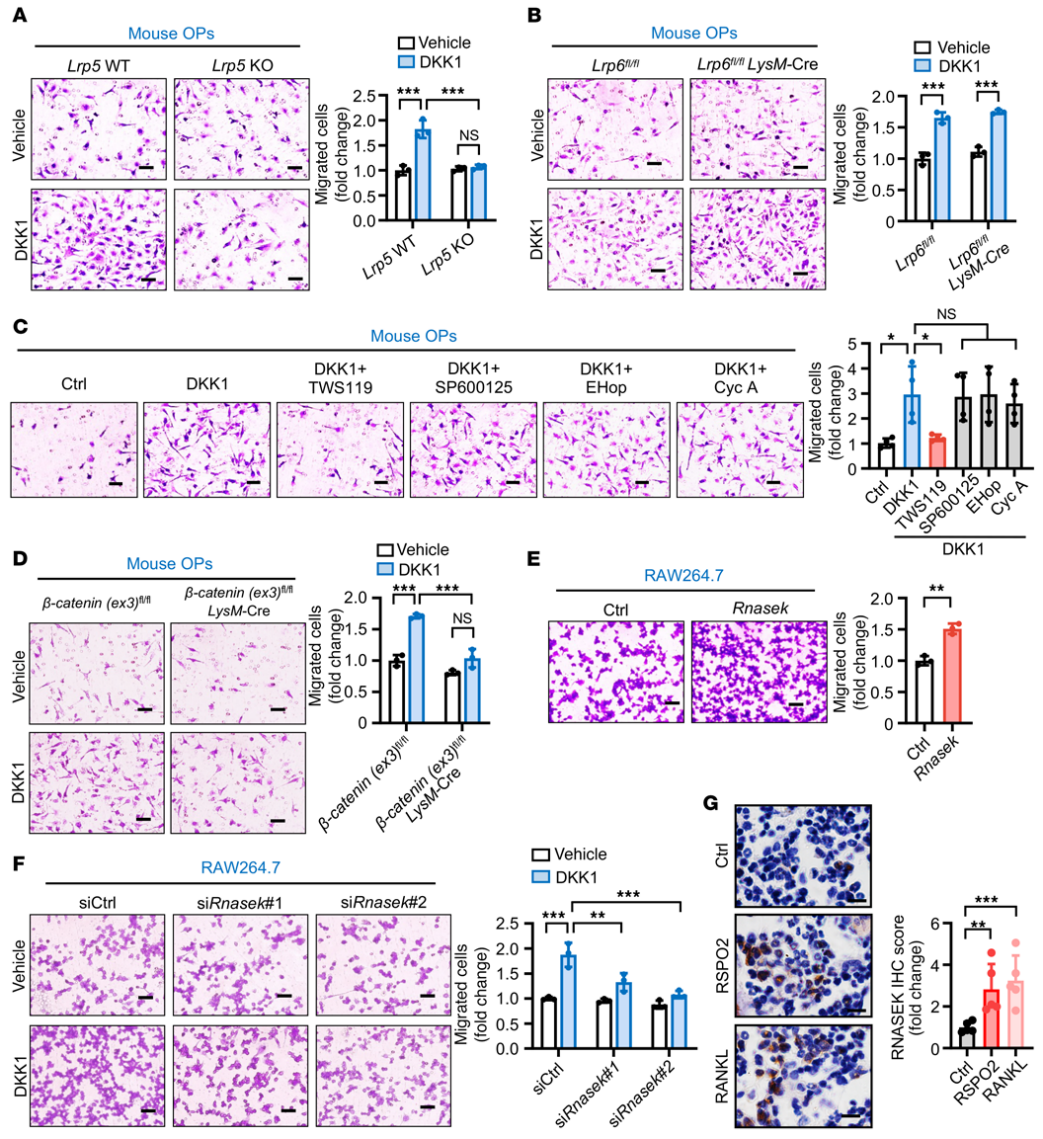
DKK1 interacted with LRP5 but not LRP6 to regulate *Rnasek* expression via canonical WNT/β-catenin signaling for OP recruitment. (**A**–**F**) Representative images are shown of *Lrp5* WT or knockout primary cultured OPs (**A**), *Lrp6^fl/fl^ LysM-*Cre primary cultured OPs (**B**), primary cultured OPs from *β-catenin(ex3)^fl/fl^* gain-of-function mutation mice (**D**), and *Rnasek*-overexpressing (**E**) and *Rnasek*-knockdown (**F**) RAW264.7 cells subjected to the Transwell migration assay; the migrated cells were counted and compared between the groups with 200 ng/mL DKK1 as chemoattractant versus vehicle. In addition, primary cultured OPs treated with the canonical WNT signaling activator (TWS119, 30 mM) or non-canonical WNT pathway signaling inhibitors (JNK inhibitor SP600125, 2 μM; Rac inhibitor EHop-016 [EHop], 8 μM; and calcineurin inhibitor cyclosporin A [Cyc A], 4 μM) were also subjected to the Transwell migration assay with or without DKK1 as chemoattractant (**C**). Quantification of the number of migrated cells is shown in the graphs. Data indicate the mean ± SD. *P < 0.05, **P < 0.01, ***P < 0.001, 1-way ANOVA with Tukey’s post hoc test (**A**, **C**, **D**, and **F**), unpaired 2-tailed Student’s *t* test (**B** and **E**). *n* = 4 biological replicates for **C** and *n* = 3 biological replicates for the others. Scale bars: 20 μm. (**G**) Representative images of IHC staining for RNASEK in tumor-free mouse tibiae (see Figure 2, B and C). Quantitative analysis of IHC staining is shown at right. Data indicate the mean ± SD. **P < 0.01, ***P < 0.001, 1-way ANOVA followed by Dunnett’s post hoc test. *n* = 5 per group. Scale bars: 10 μm.

Since DKK1 was downstream of RSPO2/RANKL-LGR4 signaling, we next sought to determine via which coreceptor, LRP5 or LRP6, DKK1 binds to regulate OP recruitment. We used 2 cell models. One involved primary cultured OPs from *Lrp5*-knockout (*Lrp5^–/–^*) or *Lrp6*-knockout (*Lrp6^flox/flox^* *LysM-*Cre) mice; the other used *Lrp5-* or *Lrp6*-silenced RAW264.7 cells. Knocking out or knocking down *Lrp5*, but not *Lrp6*, inhibited DKK1-induced OP recruitment (Figure 8, A and B, and Supplemental Figure 8, A and B), suggesting that DKK1 regulated OP recruitment by functionally binding to LRP5.

Since DKK1 regulates both canonical and non-canonical WNT signaling (28, 29), we tested whether DKK1 regulates OP recruitment through canonical or non-canonical WNT signaling. Four different canonical or non-canonical WNT signaling inhibitors or activators were used. Our data showed that only TWS119, an activator of canonical WNT/β-catenin signaling, but not the JNK inhibitor SP600125, the Rac inhibitor EHop-016, or the calcineurin inhibitor cyclosporin A, significantly inhibited DKK1-induced OP recruitment (Figure 8C) without cytotoxicity (Supplemental Figure 8C). Moreover, using primary cultured OPs from *β-catenin(ex3)^flox/flox^* gain-of-function mutation mice, β-catenin activation was shown to significantly inhibit DKK1-induced OP recruitment (Figure 8D). All of our data indicated that DKK1 regulated OP recruitment by suppressing canonical WNT/β-catenin signaling.

To determine which downstream target genes regulated by DKK1–LRP5–β-catenin signaling affected OP recruitment, we examined the transcriptome of OPs with or without DKK1 stimulation. *Zc3h11a*, *Rnasek*, *Usp49*, *Btbd6*, and *Ly6c1* were the 5 most highly upregulated genes upon treatment with DKK1 (Supplemental Figure 8D). After verification using quantitative RT-PCR, *Rnasek* was selected for further investigation (Supplemental Figure 8E). RNASEK is a transmembrane V-ATPase–associated factor that regulates the migration of multiple cell types (30–32). Therefore, we examined the function of *Rnasek* in regulating OP recruitment. Our results showed that overexpressing *Rnasek* notably enhanced OP recruitment (Figure 8E and Supplemental Figure 8F). Conversely, knocking down *Rnasek* inhibited DKK1-induced OP recruitment (Figure 8F and Supplemental Figure 8G). Consistently, in the premetastatic niche in vivo mouse model, the number of RNASEK-positive cells was increased in the RSPO2- or RANKL-stimulated groups (Figure 8G; refer to Figure 2, B and C). In conclusion, DKK1 interacted with the WNT coreceptor LRP5 to block canonical WNT signaling and to upregulate *Rnasek*, thereby attracting OPs.

### Targeting LGR4 signaling reduces BCa bone metastasis.

**Figure 9:**
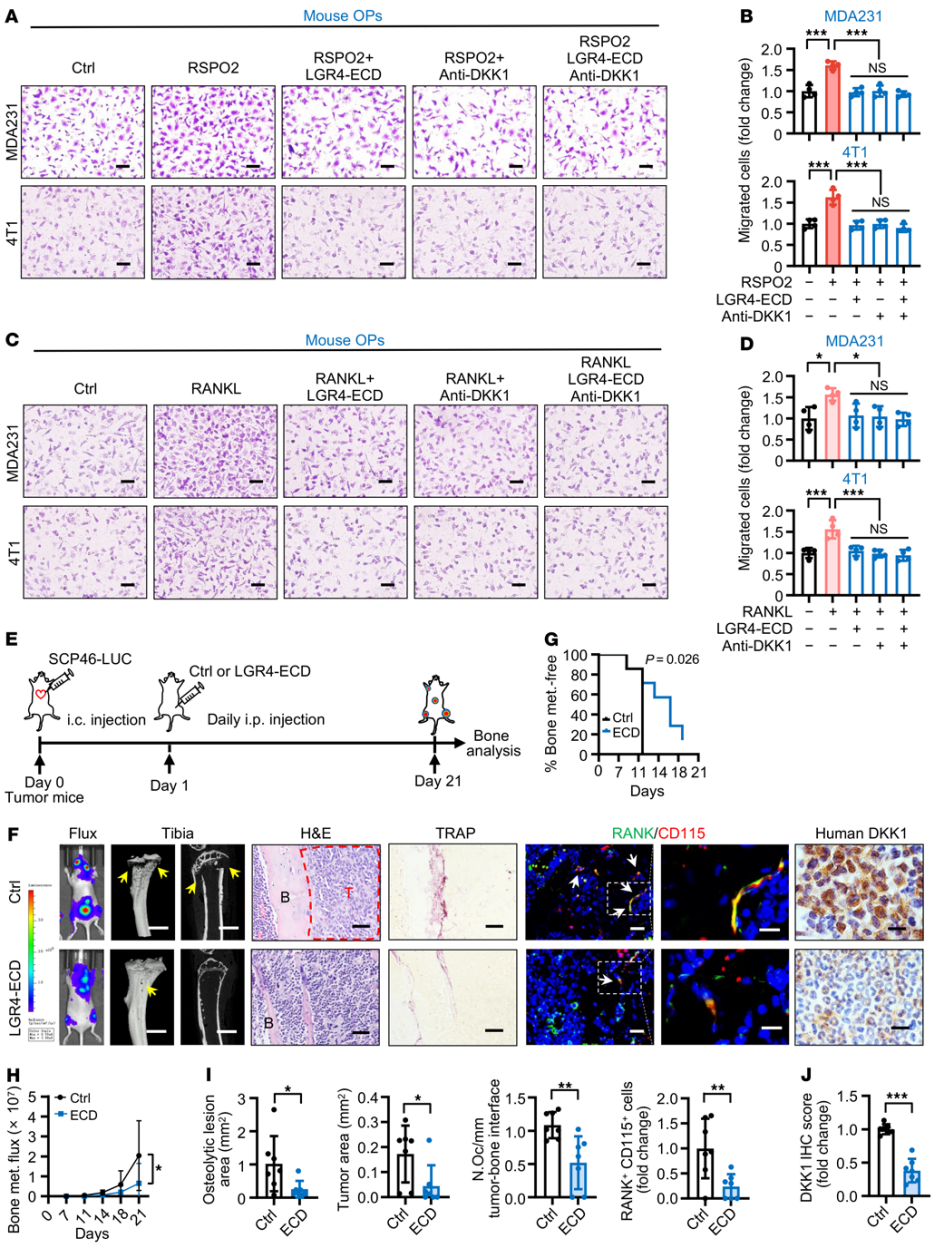
Targeting LGR4-DKK1 signaling for the treatment of BCa bone metastasis. (**A** and **B**) Representative images of mouse primary OPs (top chamber) subjected to the Transwell migration assay with conditioned medium from MDA231 (top) or 4T1 (bottom) cells treated with RSPO2 (200 ng/mL), LGR4-ECD (5.4 μg/mL), and anti-DKK1 (1 μg/mL) (**A**) with quantification of the number of migrated cells (**B**). Data indicate the mean ± SD. ***P < 0.001, 1-way ANOVA with Tukey’s post hoc test. *n* = 4 biological replicates. Scale bars: 20 μm. (**C** and **D**) Representative images of mouse primary OPs (top chamber) subjected to the Transwell migration assay with conditioned medium from MDA231 (top) and 4T1 (bottom) cells treated with RANKL (200 ng/mL), LGR4-ECD (5.4 μg/mL), and anti-DKK1 (1 μg/mL) (**C**) with quantification of the number of migrated cells (**D**). Data indicate the mean ± SD. *P < 0.05, ***P < 0.001, 1-way ANOVA with Tukey’s post hoc test. *n* = 4 biological replicates. Scale bars: 20 μm. (**E**) Experimental design of the in vivo bone metastasis mouse model. SCP46-LUC cells were injected i.c. into nude mice. After 1 day, the mice were injected i.p. daily with LGR4-ECD (1 mg/kg/d) for 21 days. (**F**–**J**) Representative images of bioluminescent, radiographic, H&E, TRAP, and IF double staining for RANK (green) and CD115 (red) and IHC staining for DKK1 in the tibiae of mice (**F**), and quantitative analysis (**G**–**J**). Yellow arrows indicate osteolytic lesions; red dotted lines indicate tumor zones; white arrows indicate cells double-positive for RANK and CD115. Data indicate the mean ± SD. *P < 0.05, **P < 0.01, ***P < 0.001, (**H**) 2-way ANOVA, (**G**) log-rank test, and (**I** and **J**) unpaired 2-tailed Student’s *t* test. *n* = 7 per group. Scale bars: 1 mm (micro-CT), 25 μm (H&E), 20 μm (TRAP), 10 μm/5 μm (left/right, IF), 10 μm (IHC).

In order to validate targeting LGR4 signaling for treatment of BCa bone metastasis, we used an anti-DKK1 antibody alone and in combination with a soluble LGR4 extracellular domain (ECD) protein (containing both the RSPO2 and the RANKL interaction domains to function as a competitive inhibitor of LGR4 binding; ref. 20) to pretreat BCa cells and assessed the effect on RSPO2- or RANKL-induced OP recruitment. The anti-DKK1 and LGR4-ECD treatments were effective in inhibiting OP recruitment in vitro, but they were not additive (Figure 9, A–D), suggesting they are likely in the same signaling pathway. Considering the effect of anti-DKK1 antibody promoting BCa lung metastasis (29), we next assessed using the soluble LGR4-ECD protein as a treatment for BCa bone metastasis in vivo. After an initial i.c. injection of SCP46 cells, a highly efficient bone tropism cell line with the highest LGR4 expression level (Figure 3C), nude mice were given daily i.p. injections of soluble LGR4-ECD protein (Figure 9E). LGR4-ECD protein treatment significantly delayed the onset of bone metastasis, alleviated SCP46-induced excessive bone metastasis burden, and ameliorated osteolytic bone lesions (Figure 9, F–I), but did not promote lung metastasis (Supplemental Figure 9A). In addition, LGR4-ECD protein treatment reduced the number of OPs and osteoclasts (Figure 9, F and I). Using a specific anti–human DKK1 antibody, we found that DKK1 from human BCa cells was significantly decreased in the LGR4-ECD protein treatment group (Figure 9, F and J, and Supplemental Figure 9B). Therefore, all our results demonstrated that LGR4 signaling can be a promising target for treating BCa bone metastasis.

## Discussion

Clinically, therapies targeting osteoclasts are extensively used for bone metastasis. However, whether and how OPs function in forming the premetastatic niche to facilitate bone metastasis are largely unknown. In this study, we found that RSPO2 and RANKL interact with LGR4 to upregulate DKK1 expression through WNT and Gα_q_ signaling in BCa cells. DKK1 protein secreted by the BCa cells circulates to bones where it attracts OPs to form a premetastatic niche by suppressing the LRP5–β-catenin–*Rnasek* axis (Supplemental Figure 10). In BCa clinical samples, RSPO2, LGR4, and DKK1 expression was significantly increased in bone metastasis patient samples compared with patient samples with primary tumor only, and there was a positive correlation in bone metastasis clinical samples between LGR4 and DKK1 expression, between RSPO2 and DKK1, and between RANKL and DKK1. Furthermore, blocking LGR4 signaling suppressed BCa bone metastasis.

It has been established that premetastatic niche formation in metastatic organs can accelerate subsequent cancer cell metastasis and colonization (1, 2). A variety of cells, most of which are immune cells, are involved in premetastatic niche formation. It has previously been shown that lysyl oxidase secreted from estrogen receptor–negative BCa cells promotes osteoclast differentiation independent of RANKL and inhibits osteoblast proliferation in the formation of premetastatic niche (9). However, subsequent research reported that lysyl oxidase failed to induce osteoclast differentiation (33), although lysyl oxidase–induced premetastatic niche formation and bone metastasis were abrogated with bisphosphonate, confirming the role of osteoclasts (9, 33). In this study, we demonstrated that recruitment of OPs is a key process in premetastatic niche formation for BCa bone metastasis. Furthermore, in addition to the known RANKL-RANK axis in bone metastasis, we present an exciting contribution of RANKL-LGR4 signaling in regulating metastatic spread to bones. This finding provides a new target for the clinical treatment of bone metastasis. The RANKL antibody denosumab and bisphosphonates are the current FDA-approved clinical drugs for bone metastases that function through inhibiting osteoclast activation and breaking the vicious cycle that promotes metastatic growth (34). However, these therapies do not improve disease-related prognosis in patients with high-risk early-stage BCa (7), suggesting that there are still other signaling pathways regulating osteoclast activity during BCa bone metastasis.

Our data show that both RANKL and RSPO2 bound to the GPCR receptor LGR4, which regulates the expression of DKK1. DKK1 was the key mediator of premetastatic niche formation, providing a possible explanation for why blocking RANKL alone is not enough to suppress bone metastasis. Although RANKL was suggested to be chemotactic for OPs (35–37), we found that RANKL has little direct chemotactic effect for OP recruitment, which is consistent with several previous studies showing that RANKL has little effect on bone marrow monocyte recruitment (38, 39). In our study, we demonstrated that knocking down the RANKL cognate receptor *RANK* in BCa cells had little effect on RANKL-stimulated *DKK1* expression and conditioned medium–induced OP recruitment. This indicates a complicated RANKL-RANK signaling in regulating bone metastasis; at a minimum, RANKL-RANK signaling in BCa cells has limited involvement in regulating DKK1-mediated osteoclastic premetastatic niche formation. Furthermore, it is reasonable to speculate that primary BCa has increased LGR4 and DKK1 levels in order to prepare the premetastatic niche in an endocrine manner. However, our data showed that LGR4 and DKK1 were both elevated in the BCa in bone metastasis tissues and correlated with each other, underlining that LGR4-DKK1 expression is positively involved in BCa bone metastasis organotropism. It is highly likely that intratumor heterogeneity (40–42) or a dichotomous effect of DKK1 on organotropic metastasis (29) has masked the possibility of capturing the enhanced DKK1 level in the primary BCa. Importantly, our in vitro data indeed provided some insights. That is when DKK1 overexpression in cell lines with either a low-efficiency (SCP6) or a high-efficiency (SCP46) bone tropism remarkably enhanced bone metastasis. Moreover, abundant data demonstrate that DKK1 can negatively regulate osteoblast differentiation in multiple cancer cell types (43, 44), suggesting that increased DKK1 in the premetastatic niche may not only inhibit osteoblastic bone formation and contribute to osteolytic lesions, but also indirectly affect OP recruitment through the bone remodeling coupling; while, without confounding factors from osteoblasts, our in vitro experiments provide strong evidence that DKK1 can recruit both primary cultured OPs (Figure 4D) and RAW264.7 cells (Supplemental Figure 5, F and G) directly in a dose-dependent manner, which was further confirmed by “rescue” recruitment experiments (Supplemental Figure 6, A and B).

Given that overexpression of DKK1 is correlated with shorter overall survival (45–47), that bone is the third most common metastasis target site for a wide range of solid tumors (48), and that several clinical trials based on anti-DKK1 neutralizing monoclonal antibodies have been done (NCT01302886, NCT01337752), it is plausible that targeting DKK1 could be effective for treating bone metastasis of solid tumors expressing DKK1. However, a report recently showed that DKK1 is a Janus-faced cytokine with dichotomous roles in cancer cell metastasis to lung and bone. DKK1 suppressed lung metastasis by regulating the lung immune microenvironment but promoted bone metastasis by targeting osteoblasts (29). Therefore, the prospect of therapies targeting DKK1 directly in treating bone metastasis is compromised by the risk of promoting lung metastasis (29). In our study, we identified the upstream regulators of DKK1 in tumors, RSPO2/RANKL and their receptor LGR4, and our results showed that soluble LGR4-ECD protein, containing both the RSPO2 and the RANKL interaction domains, significantly inhibited bone metastasis, but did not promote lung metastasis. Considering that high expression of LGR4 in BCa cells markedly promotes lung metastasis (22), LGR4-ECD could have a direct effect on BCa cells and thus possibly decrease its lung metastasis, suggesting that RSPO2/RANKL-LGR4 could be a promising target for inhibiting bone metastasis, and without side effects on lung metastasis. Our previous report showed that LGR4-ECD protein could directly inhibit osteoclast differentiation in 3 murine osteoporosis models (20). Therefore, LGR4-ECD protein could be a promising target for bone metastasis because of its dual function on both cancer cells and osteoclasts.

In our study, we found that RSPO2 or RANKL strongly stimulated DKK1 expression in luminal and basal-like BCa cell lines, while effects were relatively lower in HER2^+^ cell lines. It has been reported that HER2^+^ tumors were associated with a significantly higher rate of brain, liver, and lung metastases but a lower frequency of bone metastasis, in comparison with the highest occurrence of bone metastasis in luminal BCa (3) and the particular aggressiveness of basal-like BCa (49). Our data suggest a new explanation for the limited bone metastatic potential of HER2^+^ tumors: RSPO2/RANKL-LGR4-DKK1 signaling might be weak in HER2^+^ subtype BCa, thus reducing bone premetastatic niche formation.

RSPOs are a group of 4 secreted factors with essential roles in organ development and survival of adult stem cells as well as in cancer development (50). Human genetic studies revealed that RSPO1 is critical for ovarian development (51), and RSPO2 mutation leads to tetra-amelia syndrome including lung aplasia and a lack of limbs (18), while RSPO4 is necessary for human nail formation (52). In breast tumors, RSPO2 and RSPO3 were first identified as being involved in BCa initiation, proliferation, epithelial-mesenchymal transition, and metastasis (53). The expression of RSPO2 and RSPO4 is increased in basal and metaplastic tumors (54), while the highest levels of RSPO3 expression were detected in basal BCa (55). We provide evidence that among the 4 members, only RSPO2 enhanced the recruitment of OPs, suggesting that the 4 members of the RSPO family have tissue-specific and spatiotemporally regulated functions in regulating cancer progression. It has been reported that RSPO2 regulates hepatocellular carcinoma (56), colon cancer progression (57), osteoblast activity (58), facial morphogenesis (59), and maturation of ovarian follicles (60) by activating canonical WNT/β-catenin signaling. However, recent publications reported that RSPO2 activates WNT signaling independent of LGR4 in governing limb development (18, 61). In our results, knocking down *LGR4* almost completely abrogated RSPO2-induced DKK1 expression, suggesting that RSPO2 activates WNT signaling in a tissue- or cell type–dependent manner. Further mechanistic research is needed especially in tissues lacking LGR. Furthermore, RSPOs/LGRs are well known to function in regulation of adult stem cells and cancer stem cells, including intestinal stem cells (62) and hair follicle stem cells (63) as well as acute myeloid leukemia stem cells (64), tongue squamous cancer stem cells (65), intestinal cancer stem cells, and BCa stem cells (22). Therefore, it is interesting to speculate that BCa stem cells stimulated by RSPO2 or RANKL may regulate the osteoclastic premetastasis niche formation. The elaboration of this concept in future studies will provide new insights into the biology of cancer stem cells in regulating the premetastatic niche.

Overall, by illuminating how OPs function as premetastatic niche components to facilitate bone metastasis, our work has elucidated a critical role for RSPO2/RANKL-LGR4-DKK1 signaling in enhancing OP recruitment and osteoclastic premetastatic niche formation. Altogether, our results indicate that LGR4 signaling could be a promising target for preventing and treating BCa bone metastasis.

## Methods

### Mice.

BALB/c nude WT mice and BALB/c WT mice of 5 weeks were purchased from the Animal Center of East China Normal University (ECNU). *Lrp5^–/–^* mice (strain C57BL/6) and *Lrp6^fl/fl^* mice (strain C57BL/6) were generated by the Animal Center of ECNU. *β-Catenin(ex3)^fl/fl^* mice (strain C57BL/6) were a gift from Gang Ma at Shanghai Jiao Tong University, Shanghai. *LysM*-Cre (strain C57BL/6), MMTV-PyMT *Lgr4^fl/fl^* (strain FVB), and MMTV-PyMT *Lgr4^+/–^* (strain FVB) mice were described in previous reports (22, 66). Only female mice were used in all experiments. For primary OP isolation, 6- to 8-week-old WT mice (strain C57BL/6) were used in our study.

### Conditioned medium collection.

Conditioned medium was collected and referenced as previously described (67, 68). Briefly, BCa cells (1 × 10^6^ 4T1, 2 × 10^6^ MDA231, 2 × 10^6^ SCP6, or 2 × 10^6^ SCP46 cells) were plated in a 10 cm dish and cultured until 85% confluent (for RSPO2 or RANKL treatment, 4T1 or SCP46 cells were stimulated with 200 ng/mL RSPO2 or RANKL). Cells were washed twice with PBS and cultured for 48 hours (24 hours for 4T1 cells) in 6 mL of serum-free and phenol red–free DMEM. The conditioned medium was collected, filtered with a 0.22 μm filter to remove cell debris, and then concentrated using a Millipore protein concentrator (Amicon Ultra-15, 10 kDa). Medium was centrifuged at 3500*g* until triple the concentration. The concentrated conditioned medium was aliquoted to avoid repeated freezing and thawing and stored at –80°C for use within 3 months.

### Premetastatic niche mouse model.

For 4T1, MDA231, SCP6, or SCP46 cells, 300 μL conditioned medium or control medium (phenol red–free DMEM) was injected i.p. daily into 5-week-old BALB/c nude or BALB/C mice for 3 weeks or indicated times to form premetastatic niches. A cohort of mice were sacrificed according to the experimental protocol, and mouse serum and bones were harvested for further analysis. The remaining mice were then injected with 3 × 10^4^ 4T1-LUC, 1 × 10^5^ SCP6-LUC, or SCP46-LUC cells into the left cardiac ventricle (day 0). Bioluminescent imaging (BLI) was performed twice a week. Mice were injected with 3 mg/kg d-luciferin and imaged using Bioluminescence Imaging (Caliper Life Sciences). Bone metastatic burden (including limbs and spines) was analyzed using Living Image 3.2 software (Caliper Life Sciences). Bone metastasis–free curves were generated by observation of BLI signal in limbs and spines.

### TRAP and H&E staining.

Mouse bone tissues were fixed in 4% paraformaldehyde solution and decalcified in 0.5 M EDTA for 14 days at 4°C, with EDTA changed once a week. Then paraffin sections (at 6 μm) were stained with tartrate-resistant acid phosphatase (TRAP; MilliporeSigma, 387A-1KT) or H&E. Images of TRAP and H&E were obtained with a Leica microscope (DM4000b). Osteoclast number, osteoclast surface area, eroded surface area, tumor-bone interface, and tumor area were assessed at 0.2 to 2 mm below the growth plate by the OsteoMeasure Analysis System (Osteometrics) and ImageJ software (NIH) according to the manufacturers’ protocols. During preparation and analysis of bone tissues, investigators were blinded to specific groups.

### OP or BCa cell migration assay.

For GPCR ligand/agonist screening, MDA231 cells were seeded into 24-well plates (bottom chamber) at a concentration of 6 × 10^4^ cells per well and stimulated with 86 GPCR ligands or agonists for 24 hours. Transwell inserts (6.5 mm) with 8 μm pores were then added to the wells, and 1 × 10^5^ RAW264.7 cells were plated in the top chamber. Cells were cocultured for 16 hours to permit recruitment. For conditioned medium–induced OP migration assays, cancer cells were transfected with indicated plasmids or siRNA. Twenty-four hours after transfection, 6 × 10^4^ cancer cells were seeded into the 24-well plates and cultured for a further 24 hours. Transwell inserts were then added into the wells, and 8 × 10^4^ primary cultured OPs were seeded into the top chamber and cocultured for 16–20 hours of recruitment. For protein- or small molecule–induced OP migration assays, blank medium with RSPO2 or RANKL (200 ng/mL), 7α,25-OHC (100 nM), or DKK1 (200 ng/mL) was placed in the bottom chamber, 1 × 10^5^ primary cultured OPs or 1.2 × 10^5^ RAW264.7 cells were added to the Transwell insert top chamber, and cells were cocultured for 16–20 hours. For BCa migration assay, 1 × 10^5^ or 3 × 10^5^ primary cultured OPs were placed in the bottom chamber, 1 × 10^5^ BCa cells were added to the Transwell insert top chamber, and cells were cocultured for 8 hours. At the end of the culture period, cells on the bottom of the migrated cells were fixed and stained with 0.1% crystal violet. At least 4 fields of view per insert were photographed, and cells were counted using Image-Pro Plus 6.0 software (Media Cybernetics).

### Tissue microarray.

For BCa tissue microarray staining, anti-LGR4 and -DKK1 IHC was performed. BCa lymph node metastasis tissue array was purchased from Avilabio Inc. (DC-Bre21008a). BCa bone metastasis tissue array was obtained from Changzheng Hospital with informed patient consent and approval by the Ethics Committee. IHC scores were obtained by the multiplicative score method to evaluate protein expression as previously described (69). This system includes the intensity and the area of positive cell staining. Average intensity scores from 0 to 3 (representing no staining and weak, intermediate, and strong staining, respectively) and percentage scores from 1 to 6 (representing 1%–4%, 5%–19%, 20%–39%, 40%–59%, 60%–79%, and 80%–100%, respectively) were assigned, and then the 2 scores were multiplied to get values from 0 to 18.

### In vivo treatment of mice with recombinant LGR4-ECD protein.

Recombinant LGR4-ECD protein expression and purification were described in our previous report (20). SCP46-LUC cells (1 × 10^5^) were injected i.c. into the 8-week-old female BALB/c nude mice at day 0, and then the mice were injected i.p. with recombinant LGR4-ECD protein (2 mg/kg) or vehicle control each day for 3–5 weeks from day 1. BLI was performed twice a week, and bone metastatic burden and bone metastasis–free curves were analyzed as described above.

For further technical information, see Supplemental Methods.

### Statistics.

Data are presented as mean ± SD. The statistical significance of the data was calculated by Prism 8 software (GraphPad) and SPSS (IBM). One-way ANOVA was used for data comparison when comparing 3 or more experimental groups, with Dunnett’s post hoc test for comparing all the groups against the same control group, or Tukey’s post hoc test or least significant difference for multiple comparisons with the same *n* in all groups. The Kruskal-Wallis test was used for multiple comparisons in clinical serum samples. The unpaired 2-tailed Student’s *t* test was used to compare experiments with only 2 groups. Correlation was calculated by Pearson’s correlation analysis. Bone metastasis flux curves were analyzed by 2-way ANOVA. Bone metastasis–free survival was analyzed according to the Kaplan-Meier method, and for differences between curves, the *P* value was calculated by the log-rank test. *P* values less than 0.05 were considered significant.

### Study approval.

All mice were housed in the Animal Center of ECNU. All animal experiments were conducted in accordance with the guidelines of the Ethics Committee at ECNU. Human BCa bone metastasis samples including tissue microarray and serum samples were obtained after participants provided written informed consent, and the study was approved by the Ethics Committee of Changzheng Hospital.

## Author contributions

Z Yue conducted experiments, acquired and analyzed data, wrote the manuscript, and provided funding. XN conducted experiments, acquired and analyzed data, and wrote the manuscript. Z Yuan, QQ, WJ, LH, JG, YD, Y Liu, ZX, ZL, Z Yang, RL, XX, YG, FY, and GC acquired and analyzed data and reviewed the manuscript. XHFZ, GH, YW, and JX provided materials and reagents, acquired and analyzed data, and reviewed the manuscript. SS, AG, NW, and ML analyzed data and wrote the manuscript. Y Li provided materials/reagents, acquired and analyzed data, reviewed the manuscript, and provided funding. JL designed the studies, wrote the manuscript, and provided funding.

## Supplementary Material

Supplemental data

Supplemental data set 1

## Version 1 11/30/2021

In-Press Preview

## Version 2 01/18/2022

Electronic publication
