# Supplementary material for: RSPO2 and RANKL signal through LGR4 to regulate osteoclastic premetastatic niche formation and bone metastasis
Source: J Clin Invest. 2022 Jan 18;132(2):e144579. doi: 10.1172/JCI144579 (PMC8759794; doi:10.1172/JCI144579)
Supplement: Supplemental data [file jci-132-144579-s082.pdf]

Supplemental Figure. 1 Conditioned medium of breast cancer (BCa) cells promoted osteoclast precursor (OP) recruitment and osteoclastic pre-metastatic niche formation.

(**A**) Experimental design of the pre-metastatic niche mouse model. MDA231 cell conditioned medium was collected and i.p. injected into nude mice every day. After 2, 3, or 4 weeks, the mice were euthanized, skeletons were collected and subjected to histological analysis. i.p. injection of the cell culture blank medium into nude mice every day for 4 weeks served as the control group (Ctrl).

(**B** and **C**) (**B**) Representative images of IF double staining for RANK (green) and CD115 (red) and TRAP staining in the 3rd lumbar vertebras (L3 spine) of tumor-free mice and (**C**) quantitative analysis. White arrow indicate cells double positive for RANK and CD115. Error bars are mean ± SD. $^*P < 0.05$, $^{**}P < 0.01$, one-way ANOVA with Tukey's post hoc test. $n = 3$ per group. Scale bars, 10 μm/5 μm (top/bottom, IF), 50 μm (TRAP).

(**D**-**G**) Representative images of IF double staining for RANK (green) and CD115 (red) and TRAP staining in the (**D**) tibias, (**E**) L3 spines, (**F**) pelvis, (**G**) skull of tumor-free mice and quantitative analysis. White arrow indicate cells double positive for RANK and CD115. Error bars are mean ± SD. $^*P < 0.05$, $^{**}P < 0.01$, unpaired two-tailed Student's t test. $n = 5$ per group. Scale bars, 10 μm/5 μm (left/right, IF in **D**-**G**), 50 μm (TRAP in **D** and **E**), 25 μm (TRAP in **F** and **G**). N.oc/B.pm, Osteoclast Number/Bone Perimeter; Oc.S/BS, Osteoclast Surface/Bone Surface; ES/BS, Eroded Surface/Bone Surface.

Supplemental Figure 2 Conditioned medium of BCa cells promoted bone metastasis by recruitment of OPs and formation of an osteoclastic pre-metastatic niche.

(**A**) Representative images of MDA231 cells which have migrated through the top chamber of the Transwell system in the presence of the indicated numbers of primary cultured OPs (bottom chamber), with quantification of the number of migrated cells. Error bars are mean ± SD. *$P < 0.05$, ***$P < 0.001$, one-way ANOVA followed by Dunnett's post hoc test. $n = 3$ biological replicates. Scale bars, 20 μm.

(**B**-**E**) Mice of subject to 3 weeks pre-treatment of conditioned or control medium were analyzed 42 days after SCP46-luc cell i.c. injection (tumor-bearing mice). (**B**) Representative images of bioluminescent, radiographic, H&E, TRAP, IF double staining for RANK (green) and CD115 (red) in the tibias of mice and (**C**-**E**) quantitative analysis. Yellow arrows indicate osteolytic lesion areas, red dotted lines indicate tumor zone, white arrow indicate cells double positive for RANK and CD115. Error bars are mean ± SD. *$P < 0.05$, **$P < 0.01$, (**C**) two-way ANOVA, (**D**) log-rank test, (**E**) unpaired two-tailed Student's *t* test. $n = 6$ per group. Scale bars, 1 mm (micro-CT), 25 μm (H&E), 50 μm (TRAP), 10 μm/5 μm (left/right, IF). T, tumor; B, bone.

(**F** and **G**) (**F**) Representative images of radiographic, H&E, TRAP, IF double staining for RANK (green) and CD115 (red) in the L3 spines of mice and (**G**) quantitative analysis. Yellow arrows indicate osteolytic lesion areas, red dotted lines indicate tumor zone, white arrow indicate cells double positive for RANK and CD115. Error bars are

mean ± SD. *$P < 0.05$, **$P < 0.01$, unpaired two-tailed Student's *t* test. *n* = 6 per group. Scale bars, 1 mm (micro-CT), 25 μm (H&E), 50 μm (TRAP), 10 μm/5 μm (left/right, IF).

(**H-K**) Representative images of H&E staining in the (**H**) pelvic, (**J**) skull of tumor-bearing mice and (**I** and **K**) quantitative analysis. Error bars are mean ± SD, unpaired two-tailed Student's *t* test. *n* = 6 per group. Scale bars, 25 μm.

(**L**) Representative images of IF staining in mice. Mouse-RANK positive for OPs/osteoclasts, human-VIMENTIN positive for MDA231 cells. Pearson's correlation analysis. *n* = 8 biological replicates. Scale bars, 50 μm.

Supplemental Figure 3 R-spondin 2 (RSPO2) or RANKL promoted OP recruitment by BCa cells.

(**A**) Representative images of RAW264.7 cells subjected to the Transwell migration assay (top chamber) with RSPO2 (200 ng/mL) or RANKL (200 ng/mL) or 7a,25-OHC (100 nM) in the bottom chamber, with quantification of the number of migrated cells. Error bars are mean ± SD. **$P < 0.01$, one-way ANOVA followed by Dunnett's post hoc test. *n* = 3 biological replicates, Scale bars, 20 μm.

(**B**) Representative images of primary cultured OPs subjected to the migration assay (top chamber) with conditioned medium from three different breast cancer cell lines (MCF-7, 4T1, AT3 cells) stimulated with RSPO2 (200 ng/mL) or RANKL (200 ng/mL) in the bottom chamber, with quantification of the number of migrated cells. Error bars

are mean ± SD. *$P < 0.05$, **$P < 0.01$, ***$P < 0.001$, one-way ANOVA followed by Dunnett's post hoc test. $n = 3$ biological replicates. Scale bars, 20 μm.

(**C**) Representative images of primary cultured OPs subjected to the migration assay (top chamber) with conditioned medium from MDA231 cells stimulated with RSPO1-4 or Norrin or RANKL at 200 ng/mL in the bottom chamber, with quantification of the number of migrated cells. Error bars are mean ± SD. *$P < 0.05$, one-way ANOVA followed by Dunnett's post hoc test. $n = 3$ biological replicates. Scale bars, 20 μm.

(**D**) Quantitative analysis of metastases in tumor-bearing mouse lung, kidney and brain by ex vivo bioluminescence (mice i.p. injected with conditioned medium of SCP46 cells treated with RSPO2 (200 ng/mL) or RANKL (200 ng/mL)). Error bars are mean ± SD, one-way ANOVA Tukey's post hoc test. $n = 6$ per group.

Supplemental Figure 4 LGR4-defiency in breast cancer cells inhibits OP recruitment.

(**A**) Kaplan-Meier analysis of bone metastasis-free survival according to *LGR5* mRNA expression data in 77 patients with BCa (acquired from microarray analysis GEO # GSE2603, *LGR5* low: $n = 38$, *LGR5* high: $n = 39$). *P* values were calculated by the log-rank test.

(**B**) MDA231 cells were transfected with the indicated siRNAs, and levels of *RANK* and *LGR4* mRNA were determined by qRT-PCR. **$P < 0.01$, ***$P < 0.001$, one-way ANOVA with Dunnett's post hoc test.

(**C**) Representative images of primary cultured OPs subjected to the Transwell migration assay (top chamber) with different MDA231 cells following treatment with or without RANKL for 24 hours (bottom chamber) with quantification of the number of migrated cells. Error bars are mean ± SD. $^{**}P < 0.01$, $^{***}P < 0.001$ by one-way ANOVA with Tukey's post hoc test. $n = 3$ biological replicates. Scale bars, 20 μm.

(**D**) Representative images of primary cultured OP cells (top chamber) subjected to the Transwell migration assay with MDA231 or BT549 cells (with or without *LGR4* knockdown or re-expression, as indicated) cultured in the bottom chamber, with quantification of the number of migrated cells. Error bars are mean ± SD. $^{**}P < 0.01$, $^{***}P < 0.001$, one-way ANOVA with Tukey's post hoc test. $n = 3$ biological replicates, Scale bars, 20 μm.

(**E**) Representative images of primary cultured OPs subjected to the Transwell migration assay (top chamber) with conditioned medium from indicated *LGR4* knockdown/half insufficient BCa cells (MDA231 and PyMT) in the bottom chamber. The migrated OPs were counted (right). Error bars are mean ± SD. $^{**}P < 0.01$, $^{***}P < 0.001$, unpaired two-tailed Student's *t* test. Scale bars, 20 μm. $n = 3$ for MDA231 cells and $n = 4$ for primary cultured PyMT cells.

(**F**) Representative images of primary cultured OPs subjected to the Transwell migration assay (top chamber) with conditioned medium from indicated *LGR4* overexpressing BCa cells (MCF-7) in the bottom chamber, with quantification of the number of migrated cells. Error bars are mean ± SD. $^{**}P < 0.01$, unpaired two-tailed Student's *t* test. $n = 5$ biological replicates. Scale bars, 20 μm.

Supplemental Figure 5 LGR4 regulates DKK1 expression in different breast cancer cells and DKK1 can recruit OPs and mildly induces osteoclast differentiation at high concentration.

(**A**) RT-PCR showing the *LGR4* and *DKK1* expression level of *LGR4* knockdown in three different BCa cell lines.

(**B**) mRNA levels of *DKK1* and *LGR4* in SCP46 Ctrl and *LGR4* knockdown cells. n = 3 biological replicates. Error bars are mean ± SD. ***P < 0.001, one-way ANOVA followed by Dunnett's post hoc test.

(**C**) Representative IHC images (DKK1) in BCa patients and quantitative analysis. Error bars are mean ± SD, unpaired two-tailed Student's t test. Primary tumors (n = 50), lymph node metastasis (n = 50). Scale bars, 20 μm.

(**D**) Correlation of *LGR4* expression with *DKK1* expression (GEO # GSE14244, GSE16554). Pearson's correlation analysis.

(**E**) Correlation of *LGR4* expression with *DKK1* expression in eight different BCa cell lines by qRT-PCR. Pearson's correlation analysis.

(**F** and **G**) (**F**) Representative images of RAW264.7 cells subjected to the Transwell migration assay (top chamber) with indicated concentrations of DKK1 added to the bottom chamber, with (**G**) quantification of the number of migrated cells. Error bars are mean ± SD. *P < 0.05, ***P < 0.001, one-way ANOVA followed by Dunnett's post hoc test. n = 3 biological replicates. Scale bars, 20 μm.

(**H** and **I**) (**H**) Representative TRAP staining images (left) of primary cultured OPs challenged with DKK1 and M-CSF (10 ng/mL) and RANKL (50 ng/mL). (**I**) The osteoclast numbers were counted. One-way ANOVA followed by Dunnett's post hoc test. *n* = 3 biological replicates. Scale bars, 50 μm.

(**J**-**M**) Conditioned medium from SCP6 cells was i.p. injected into nude mice for 21 days followed by SCP6-LUC i.c. injected (tumor-bearing mice). (**J**) Representative images of bioluminescent, radiographic, H&E, TRAP, IF double staining for RANK (green) and CD115 (red) in the tibias of mice and (**K**-**M**) quantitative analysis. Yellow arrows indicate osteolytic lesion areas, red dotted lines indicate tumor zone, white arrow indicate cells double positive for RANK and CD115. (**K**) *DKK1* mRNA level in SCP6 following overexpression of *DKK1*. Error bars are mean ± SD. **P* < 0.05, ***P < 0.001, (**K** and **M**) unpaired two-tailed Student's *t* test, (**L**) two-way ANOVA and log-rank test. (**K**) *n* = 3 biological replicates, (**L** and **M**) *n* = 6 biological replicates. Scale bars, 1 mm (micro-CT), 25 μm (H&E), 50 μm (TRAP), 10 μm/5 μm (left/right, IF).

Supplemental Figure 6 Knocking down *DKK1* suppresses the capability of *LGR4* overexpressing cancer cells to attract OPs.

(**A**) Representative images of primary cultured OP cells (top chamber) subjected to the Transwell migration assay. MDA231 cells with or without *LGR4* knocked down or exogenous expression of *DKK1* were placed in the bottom chamber, with quantification of the number of migrated cells. Error bars are mean ± s.d. ****P* < 0.001 by one-way ANOVA with Tukey's post hoc test. *n* = 3 biological repeats. Scale bars, 20 μm.

(**B**) Representative images of primary cultured OPs subjected to the Transwell migration assay with conditioned medium from MCF-7 cells transfected with *LGR4* overexpression plasmid and/or si*DKK1* in the bottom chamber, with quantification of the number of migrated cells. Error bars are mean ± SD. ***P < 0.001, one-way ANOVA with Tukey's post hoc test. n = 3 biological replicates. Scale bars, 20 μm.

(**C**) Serum human DKK1 protein levels were examined using a Human DKK1 ELISA Kit in tumor-free mice after 21 days of pre-treatment. Error bars are mean ± SD. *P < 0.05, **P < 0.01, one-way ANOVA with LSD test. n = 6 per group.

(**D**) *LGR4* and *DKK1* mRNA levels in SCP46 cells following knockdown of *LGR4* by two shRNAs or overexpression of *DKK1*. Error bars are mean ± SD. **P < 0.01, *** P < 0.001, one-way ANOVA with Tukey's post hoc test. n = 3 biological replicates.

(**E**) Quantitative analysis of TRAP staining in the tibias of mice (refer to Figure 5**A**, tumor-free mice). Error bars are mean ± SD. **P < 0.01, ***P < 0.001, one-way ANOVA with Tukey's post hoc test. n = 6 per group.

(**F**) IF double staining for RANK (green) and CD115 (red) in the tibias of mice (refer to Figure 5**C**, tumor-bearing mice). Error bars are mean ± SD. *P < 0.05, ***P < 0.001, one-way ANOVA with Tukey's post hoc test. n = 6 per group.

(**G** and **H**) (**G**) Representative images of IF double staining for RANK (green) and CD115 (red) and TRAP staining in tibias of tumor-free mice. (**H**) Quantitative analysis of the bone parameters from tumor-free mice. White arrow indicate cells double positive for RANK and CD115. Error bars are mean ± SD. *P < 0.05, **P < 0.01, ***P < 0.001, one-way ANOVA Tukey's post hoc test. n = 5 per group. Scale bars, 10 μm/5

μm (left/right, IF), 50 μm (TRAP). *DKK1* mRNA levels in SCP46 cells following knockdown of *DKK1*. Error bars are mean ± SD. *** $P < 0.001$, unpaired two-tailed Student's *t* test. $n = 3$ biological replicates.

(**I**) Quantitative analysis of TRAP in the tibias of mice (refer to Figure 6**A**, tumor-free mice). Error bars are mean ± SD. *$P < 0.05$, ***$P < 0.001$, one-way ANOVA with Tukey's post hoc test. $n = 5$ per group.

(**J**) IF double staining for RANK (green) and CD115 (red) in the tibias of mice (refer to Figure 6**C**, tumor-bearing mice). Error bars are mean ± SD. **$P < 0.01$, one-way ANOVA with Tukey's post hoc test. $n = 6$ per group.

Supplemental Figure 7 RSPO2 and RANKL regulates the expression of DKK1 in different types of BCa cells.

(**A** and **B**) Correlation between serum level of RSPO2 and DKK1, RANKL and DKK1, and RANKL and RSOP2 in human benign lump patients ($n = 9$, **A**) and primary tumor patients ($n = 28$, **B**). Pearson's correlation analysis.

(**C**) mRNA levels of *DKK1* in different types of BCa cells stimulated with RSPO2 (200 ng/mL) or RANKL (200 ng/mL) for 6 hours. Error bars are mean ± SD. *$P < 0.05$, **$P < 0.01$, ***$P < 0.001$, unpaired two-tailed Student's *t* test. $n = 5$ (MDA231 cells) and $n = 3$ (other cell lines) biological replicates.

(**D**) *Dkk1* mRNA levels in 4T1 cells stimulated with RSPO2 (100 ng/mL) and RANKL (200 ng/mL) for 6 hours. Error bars are mean ± SD. *$P < 0.05$, ***$P < 0.001$, unpaired two-tailed Student's *t* test. $n = 3$ biological replicates.

(**E**) DKK1 protein levels in MDA231 cells after treatment with RSPO2 (200 ng/mL) or RANKL (200 ng/mL) for 6 hours, as determined by ELISA assay. Error bars are mean ± SD. ****P* < 0.001, unpaired two-tailed Student's *t* test. *n* = 3 biological replicates.

(**F**) DKK1 protein levels in 4T1 cells when treated with RSPO2 (200 ng/mL) and RANKL (200 ng/mL) for 24 hours.

(**G**) Mouse DKK1 protein levels in the mouse serum of the pre-metastatic niche mouse model (refer to Fig 2**, B** and **C**). Error bars are mean ± SD. **P* < 0.05, one-way ANOVA followed by Dunnett's post hoc test. *n* = 5 biological replicates.

(**H**) *RANK* mRNA levels in MDA231 cells following knockdown of *LGR4* or *RANK* or both. Error bars are mean ± SD. ****P* < 0.001, one-way ANOVA with Tukey's post hoc test. *n* = 3 biological replicates.

Supplemental Figure 8 DKK1 regulates *Rnasek* expression via canonical WNT signaling for OP recruitment.

(**A**) *Lrp5* or *Lrp6* mRNA levels in RAW264.7 cells following *Lrp5* or *Lrp6* knockdown, compared to control cells. Error bars are mean ± SD. ****P* < 0.001, unpaired two-tailed Student's *t* test. *n* = 3 biological replicates.

(**B**) Representative images of *Lrp5* or *Lrp6* knockdown RAW264.7 cells subjected to the Transwell migration assay and the migrated cells were counted and compared between the group with 200 ng/mL DKK1 as chemo-attractant and vehicle. Quantification of the number of migrated cells (right). Error bars are mean ± SD. ****P*

< 0.001, one-way ANOVA with Tukey's post hoc test. $n = 3$ biological replicates. Scale bars, 20 μm.

(**C**) Effects of TWS119 on OP viability for 24 hours. Error bars are mean ± SD. unpaired two-tailed Student's *t* test. $n = 3$ technical replicates.

(**D**) A heat map of RNA-Seq data indicating top 6 most upregulated genes ($P \leq 0.01$) in primary cultured OPs treated with DKK1 (200 ng/mL) compared to the untreated control, $n = 2$ per group. The color code scale represents Z-score values of the mean expression levels of genes

(**E**) Transcript levels of top 5 upregulated genes (refer to Supplemental Figure 8**D**) in primary cultured OPs treated with DKK1 (200 ng/mL) compared to the untreated control. Error bars are mean ± SD. $*P < 0.05$, unpaired two-tailed Student's *t* test. $n = 3$ biological replicates.

(**F**) *Rnasek* mRNA levels in RAW264.7 cells transfected with mouse *Rnasek* expression plasmid. Error bars are mean ± SD. $**P < 0.01$, unpaired two-tailed Student's *t* test. $n = 3$ biological replicates.

(**G**) *Rnasek* mRNA levels in RAW264.7 cells treated with two si*Rnasek*. Error bars are mean ± SD. $***P < 0.001$, one-way ANOVA followed by Dunnett's post hoc test. $n = 3$ biological replicates.

Supplemental Figure 9 LGR4 extracellular domain (ECD) protein treatment has little effect on lung metastasis in BCa bone metastasis mouse model.

(**A**) Representative bioluminescent images of lung in LGR4-ECD treated mice (left, refer to Figure 9, **E-G)**. Quantitative analysis of metastatic cells in lung by bioluminescence (right). Error bars are mean ± SD, unpaired two-tailed Student's *t* test. $n = 7$ per group.

(**B**) Representative images of IHC staining for human-DKK1 in tibias of tumor-bearing model from SCP46-LUC injected BALB/C nude mice (refer to Figure 9, **E-G**, $n = 7$) or 4T1-LUC injected BALB/C mice (refer to Figure 2, **D-G***,* $n = 6$) with quantitative analysis of IHC staining. Error bars are mean ± SD. ***$P < 0.001$. Scale bars, 10 μm.

Supplemental Figure 10 Working model of RSPO2/RANKL-LGR4-DKK1 axis regulating osteoclastic pre-metastatic niche formation.

RSPO2 and RANKL interact with their receptor LGR4 to modulate the expression of DKK1 through Gαq and β-catenin signaling in BCa cells. In OPs, DKK1 functionally interacts with LRP5 and regulates the expression of *Rnasek* via canonical WNT signaling for OP recruitment.

Supplemental Methods

Cell culture and reagents

MDA231, MDA468, BT549, BT474, MCF7, KPL-4, 4T1 and RAW264.7 cell lines were obtained and authenticated by the Chinese Academy of Sciences Committee Type Culture Collection Cell Bank (Shanghai, China). The cell lines were cultured according to manufacturers' instructions. MDA231 subline SCP6, MDA231 subline SCP46 cells were derived by in vivo selection and single-cell subcloning from the parental MDA231 cell line (1, 2). SCP6, SCP46 and SKBR3 cells were kindly gifts from Dr. Guohong Hu at Chinese Academy of Sciences, Shanghai and cultured in DMEM containing 10% FBS. AT3 cells were a kindly gift from Dr. Xiang H.-F. Zhang at Baylor College of Medicine and cultured in RPMI-1640 with 10% FBS. Primary cultured OPs were isolated from the femur and tibia of 6-8-week-old mice and cultured as previously described (3), the primary OPs were cultured for 4 days with mouse M-CSF treatment (10 ng/mL) after which they were used to perform all cell experiments. Chemicals and recombinant proteins were purchased as follows: FH535 (HY-15721; MedChemExpress), TWS119 (HY-10590; MedChemExpress), SP600125 (S1460; Selleckchem), EHop-016 (S7319, Selleckchem), Cyclosporin A (S2286, Selleckchem), mouse RSPO2 (6946-RS-025/CF, R&D), human RANKL (6449-TEC-010/CF, R&D), mouse RANKL (462-TEC/CF, R&D), mouse M-CSF (416-ML-050, R&D), mouse DKK1 (5897-DK-010, R&D). Antibodies to c-Fms/CD115 (ab254357) and DKK1 (ab109416 for anti-human DKK1) were obtained from Abcam; antibodies against alpha Tubulin (GB11200) were obtained from Servicebio; anti-RNASEK Antibody (NBP1-

92338) was obtained from Novusbio; monoclonal antibodies against RANK (sc-374360 for tumor-free model), RANK (sc-390655 for tumor-bearing model) and DKK1 (sc-374574 for neutralizing DKK1) were obtained from Santa Cruz Biotechnology. Anti-LGR4 Antibody was a kind gift from Dr. Qingyun Liu at University of Texas Health Science Center, Houston, TX.

Micro-CT analysis

Bone was scanned by in vivo X-ray microtomography (1272 Skyscan, Bruker) at 60 kV with a 0.25 μm aluminum filter using a detection pixel size of 9 μm, and the scanned images were reconstructed using Skyscan Recon software (Bruker) according to the manufacturer's instructions. The reconstructed images were analyzed using DataViewer software (Bruker) and CTVox software (Bruker). Osteolytic lesion area was analyzed using the ImageJ software (National Institutes of Health).

IHC staining

Mouse bone tissues were fixed in 4% paraformaldehyde solution, decalcified in 0.5 M EDTA for 14 days at 4°C, with EDTA changed once a week. For antigen retrieval of paraffin (6 μm) sections, heating at 98°C water bath for 10 minutes was performed. The paraffin sections were treated with 3% H2O2 for 10 minutes then permeabilized with 0.1% Triton X-100 (Sigma) and blocked in 2% goat serum, then incubated with LGR4, DKK1 or RNASEK antibody at 4°C overnight. Secondary antibody incubation was performed at room temperature for 1 hour, followed by detection with ABC-HRP Kit

(Vector, PK-4001) and DAB substrate (Vector, SK-4100). IHC images were obtained with a Leica microscope (Leica, DM4000b). DAB positive score analysis was performed using ImageJ software with IHC Profiler. To preclude nonspecific signals, a negative control lacking primary Ab was added with each batch of slides. During preparation and analysis of bone tissues, investigators were blinded to specific groups.

Osteoclast differentiation assay

For DKK1-induced osteoclast differentiation analysis, we isolated OPs from 6-8-week-old WT mice femur and tibia bones as previously described (3). OPs were seeded into 96-well plates at a concentration of 8000 cells per well in triplicate. Cells were stimulated with osteoclast differentiation medium (containing 50 ng/mL RANKL and 10 ng/mL M-CSF) and indicated concentrations of DKK1 for 4-6 days until differentiated. Osteoclasts were fixed and stained using the TRAP staining kit (Sigma-Aldrich, 387A-1KT). TRAP positive cell numbers were quantified and normalized to control.

Gene knockdown preparation and transfection

The non-targeting control shRNA, two *LGR4* shRNA sequences, non-targeting control siRNA, two human *LGR4* siRNA oligos were described in our previous report (4). Human *RANK* siRNA oligos (si*RANK*#1 (5); si*RANK*#2, 5'-UCUCUGCCAGCUAGAAAACUU-3') were obtained from GenePharma (Shanghai, China). Human *DKK1* shRNA sequence were used as previously described (6). Human

Gaq siRNA oligos (si*Gaq*#1, 5'-GAAGGUGUCUGCUUUUGAGTT-3'; si*Gaq*#2, 5'-GGAGUACAAUCUGGUCUAAUU-3'). Human *DKK1* siRNA oligos were also described in a previous publication (7). Mouse *Lrp5* siRNA oligos (si*Lrp5*, 5'-GGACGGACUCCGAGACCAAdTdT-3'), mouse *Lrp6* siRNA oligos (si*Lrp6*, 5'-AUCUCAACCAGAUGUGUAUUU-3') (8) and mouse *Rnasek* siRNA oligos (si*Rnasek*#1, 5'-UCCUCAGAACAUAUACAACCUGU-3'; si*Rnasek*#2, 5'-UGUCCAUUCUGCUGUGUUAAU-3') were obtained from Biotend (Shanghai, China). Breast cancer cells were transfected with siRNAs using Lipofectamine 2000 (Thermo Fisher, 11668019), and the OPs were transfected with plasmids or siRNAs using FuGENE® HD Transfection Reagent (Promega, E2311).

Gene expression analysis

For RT-PCR or qRT-PCR analysis, total RNA was extracted from cells using TRIzol reagent (MaGen, R4801-02). cDNA was generated by using PrimeScript^{TM} RT Reagent Kit (Takara, Code No. RR037A) following the manufacturer's protocol. Primers are provided in Supplemental Table 7.

Proteomic profiling of cell cultured medium

MDA468 WT and *LGR4* CRISPR KO cells (CRISPR guide sequences were as follows: sg*LGR4*#1: 5'-CTGCGACGGCGACCGTCGGG-3'; sg*LGR4*#2: 5'-GGGCTGACGGCCGTGCCCGA-3'; sg*LGR4*#3: 5'-TACCCAGTGAAGCCATTCGA-3') were cultured with serum-free media 24 h before

the growth media were collected. The media was centrifuged at 16,000 g for 10 min to remove cells and debris. The supernatant was subsequently digested with trypsin. The resulting peptides were analyzed on a Q Exactive Plus mass spectrometer and quantified by the label-free method. The values shown in the Supplemental Table 5 are in fraction of total (FOT) (iBAQ/total iBAQ × 10^6).

Identification of secreted proteins

We extracted the secreted proteins from the Uniprot database using the following two methods. First, the proteins annotated as secreted for the subcellular localization were selected. Then, the proteins from the Gene Ontology (GO) terms of proteinaceous extracellular matrix (GO: 0005578) and extracellular space (GO: 0005615) as well as their child nodes were added as well. In total, 2,090 secreted proteins were obtained (listed in Supplemental Table 3).

CCK-8 assay

The CCK-8 assay was performed to examine cell viability. OPs (20000/well) were seeded in 96-well plates with OPs culture medium (100 μl), three replicate wells per group. Cells were treated with or without TWS119 in the medium as indicated. Finally, 10 μl of CCK-8 reagent were added to each well and incubated for 1 - 3 hours. Then the absorbance value (OD) was measured.

RNA-Seq and bioinformatics analysis

Primary cultured OPs isolated from two mice were seeded in 6-well plates in 2 ml OP culture medium per well. The cells were stimulated with or without DKK1 (200 ng/mL) for 3 hours. Total cellular RNA was extracted with TRIzol (MaGen, R4801-02). The concentration and purity of the RNA samples were examined using an Agilent 2100 Bioanalyzer and a NanoDrop 2000 Spectrophotometer. The edgeR package (http://www.bioconductor.org) was used to analyze the differential gene expression of the two groups. Genes with adjusted P <0.05 and absolute log2 [fold change] > 1 were considered to be significantly differentially expressed. The RNA-Seq data have been deposited in the NCBI Gene Expression Omnibus database under the accession number GSE184248.

Immunofluorescence (IF) staining

Mouse bone tissues were fixed in 4% paraformaldehyde solution, decalcified in 0.5 M EDTA for 14 days at 4°C, with EDTA changed once a week. Protease K (sigma) was used for antigen retrieval of paraffin sections (6 μm). The paraffin sections were permeabilized with 0.1% Triton X-100 (Sigma) and blocked in 2% goat serum, then incubated with CD115 (also known as c-Fms/CSF-1R) and RANK antibody at 4ºC overnight. Incubation with fluorescently labeled secondary antibody was performed at room temperature for 1 hour followed by mounting medium with DAPI (Invitrogen, P36962). Images were obtained with Leica microscope (Leica, DM4000b). The number of positively stained cells were counted in the specimen per mouse. To preclude nonspecific signals, a negative control lacking primary antibody was included with each

batch of slides. During preparation and analysis of bone tissues, investigators were blinded to specific groups.

Serum ELISA analysis

47 serum samples of benign breast lump, primary BCa, and bone metastasis BCa patients were obtained from Changzheng Hospital of The Second Military Medical University with informed patient consent and the approval by the Ethics Committee. Serum samples were pre-treated according to the manufacturer's instructions and analyzed with human DKK1 ELISA kit (SEA741Hu, Cloud-Clone), human RSPO2 ELISA kit (SEA172Hu, Cloud-Clone) and human RANKL ELISA kit (SEA855Hu, Cloud-Clone) according to the standard protocol.

Reference

1. Kang Y, et al. A multigenic program mediating breast cancer metastasis to bone. *Cancer cell.* 2003;3(6):537-49.
2. Minn AJ, et al. Genes that mediate breast cancer metastasis to lung. *Nature.* 2005;436(7050):518-24.
3. Takeshita S, et al. Identification and characterization of the new osteoclast progenitor with macrophage phenotypes being able to differentiate into mature osteoclasts. *Journal of bone and mineral research : the official journal of the American Society for Bone and Mineral Research.* 2000;15(8):1477-88.
4. Yue Z, et al. LGR4 modulates breast cancer initiation, metastasis, and cancer stem cells. *FASEB journal : official publication of the Federation of American Societies for Experimental Biology.* 2018;32(5):2422-37.
5. Luo J, et al. LGR4 is a receptor for RANKL and negatively regulates osteoclast differentiation and bone resorption. *Nat Med.* 2016;22(5):539-46.
6. Zhuang X, et al. Differential effects on lung and bone metastasis of breast cancer by Wnt signalling inhibitor DKK1. *Nature cell biology.* 2017;19(10):1274-85.
7. Hall CL, et al. p21CIP-1/WAF-1 induction is required to inhibit prostate cancer growth elicited by deficient expression of the Wnt inhibitor Dickkopf-1. *Cancer Res.* 2010;70(23):9916-26.

8. Wei W, et al. The LDL receptor-related protein LRP6 mediates internalization and lethality of anthrax toxin. *Cell.* 2006;124(6):1141-54.

Supplemental Figure 1

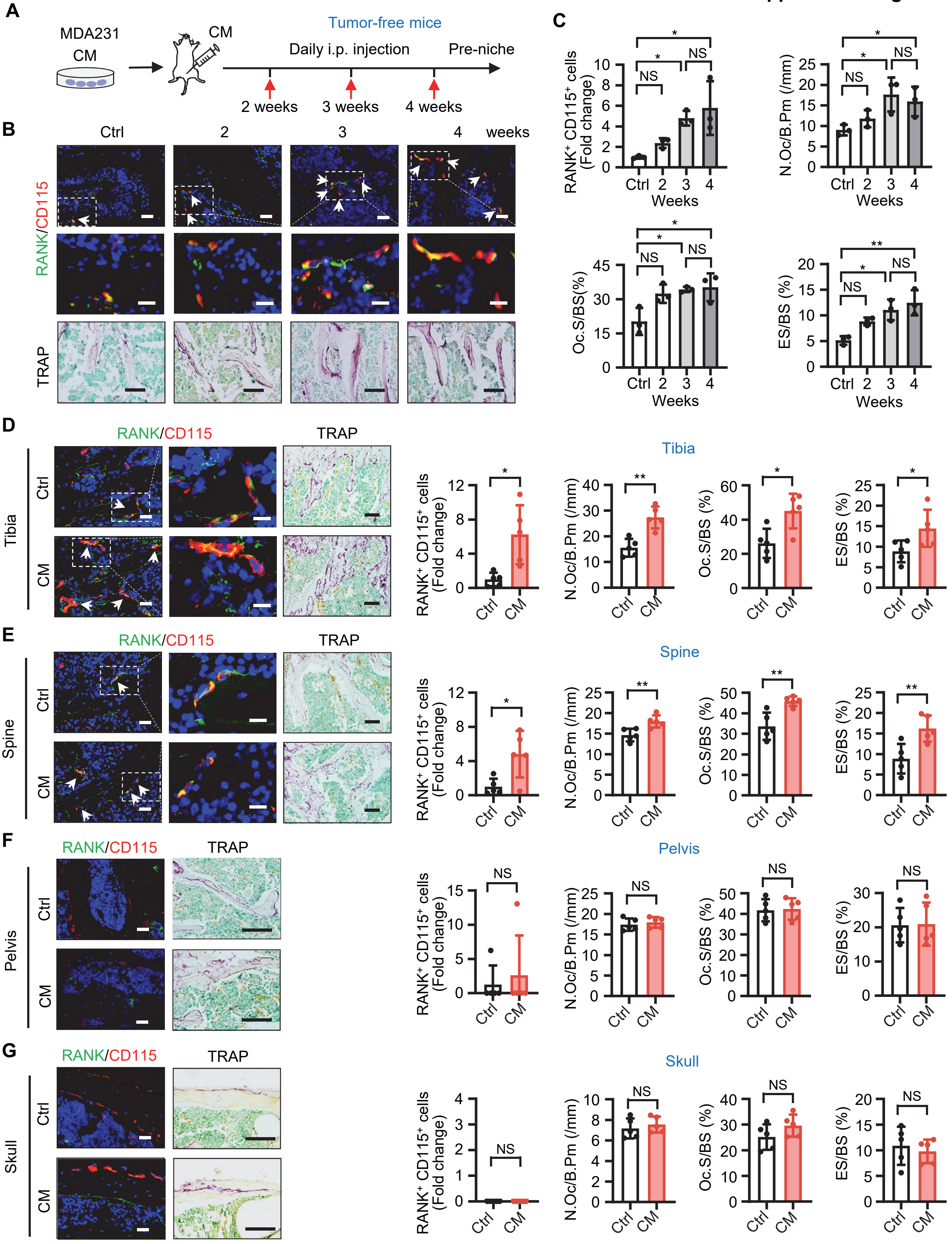

Supplemental Figure 2

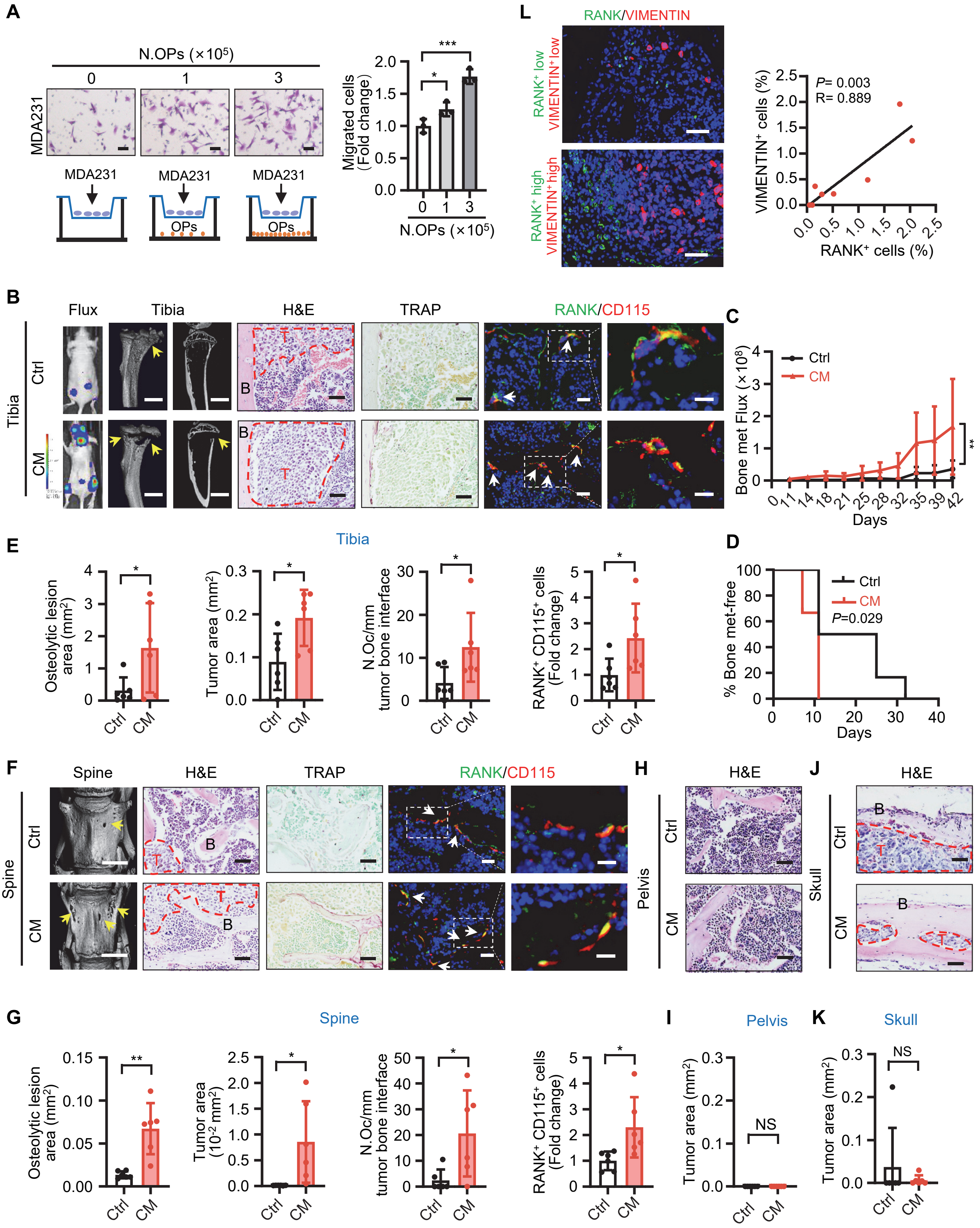

Supplemental Figure 3

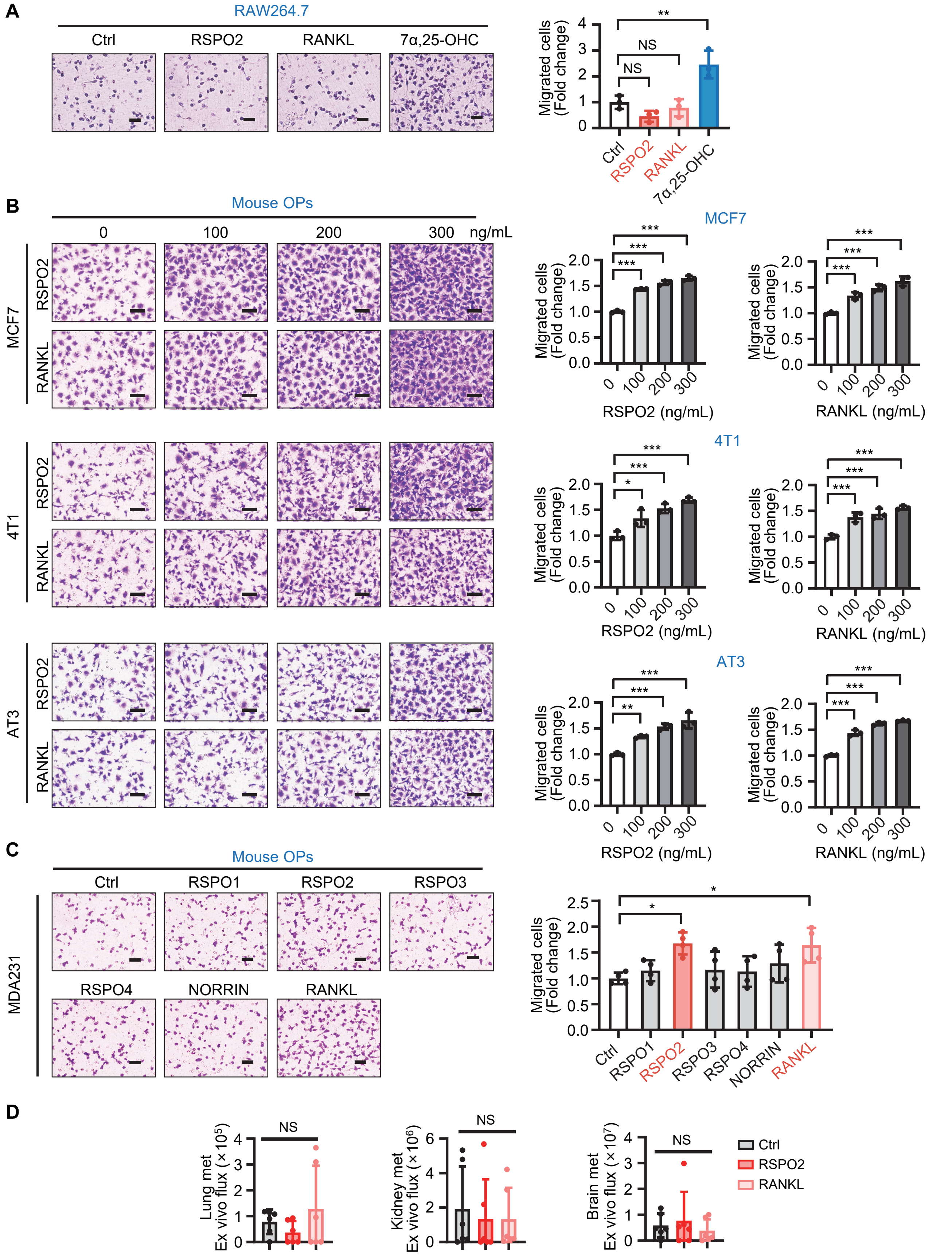

Supplemental Figure 4

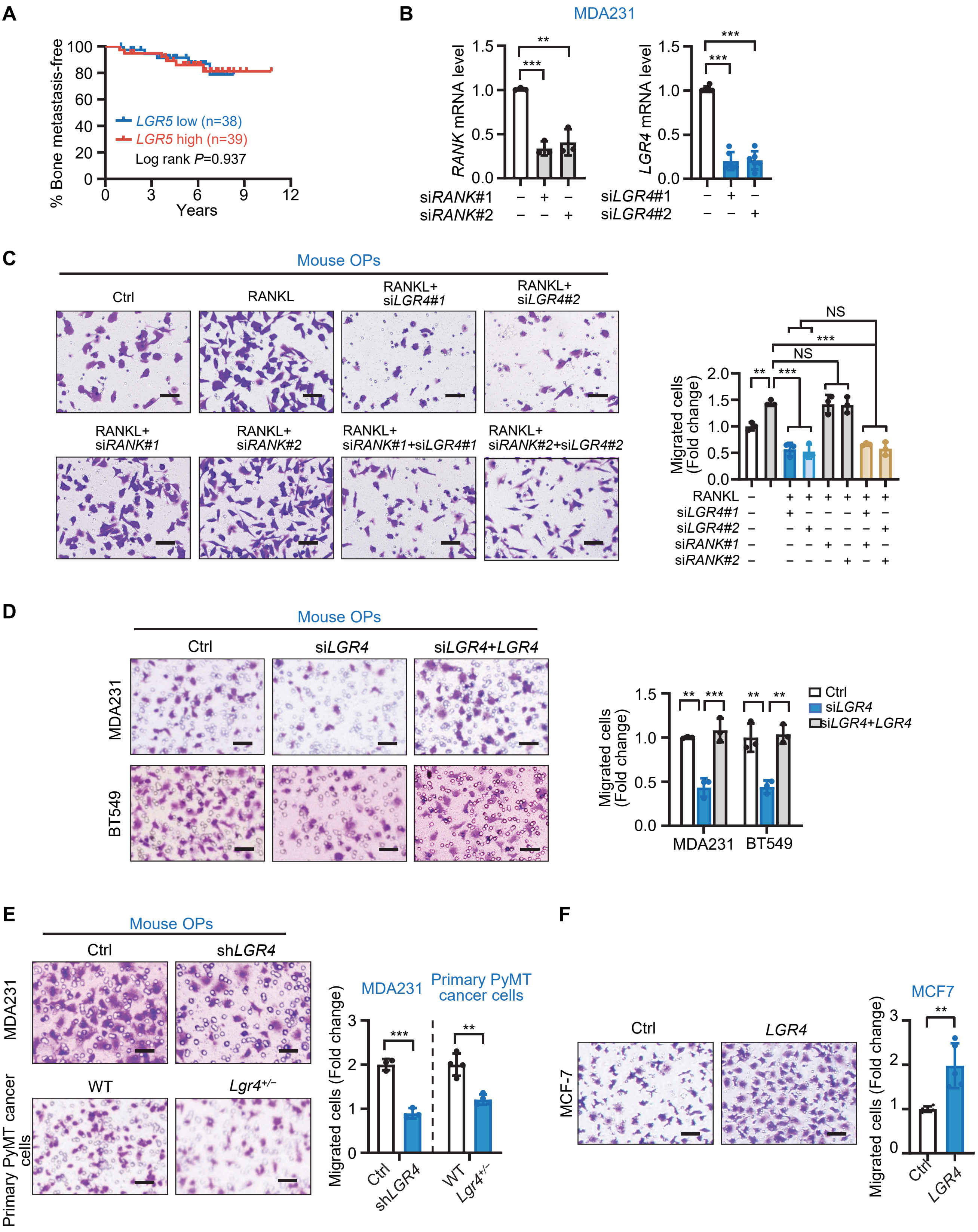

Supplemental Figure 5

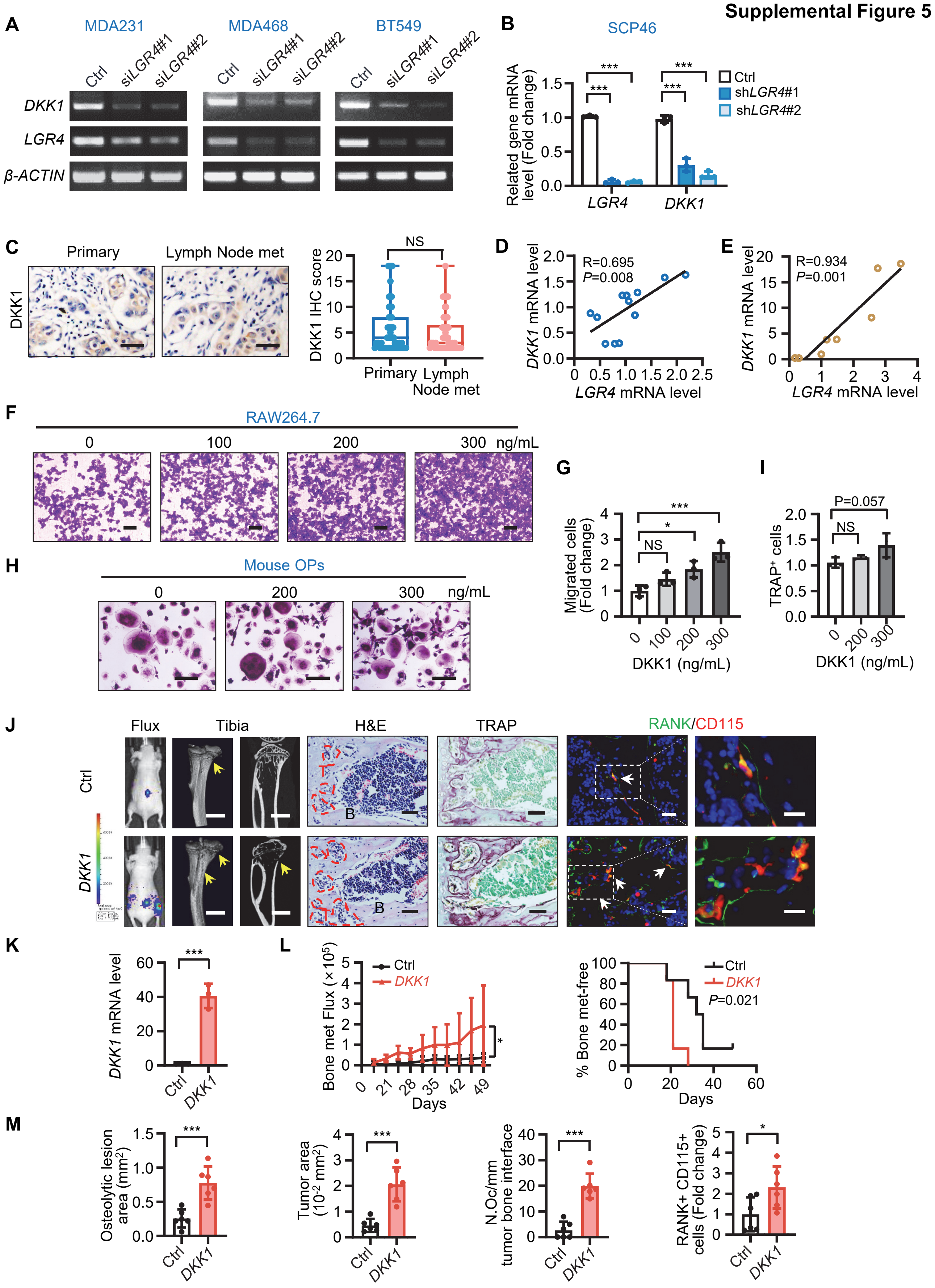

Supplemental Figure 6

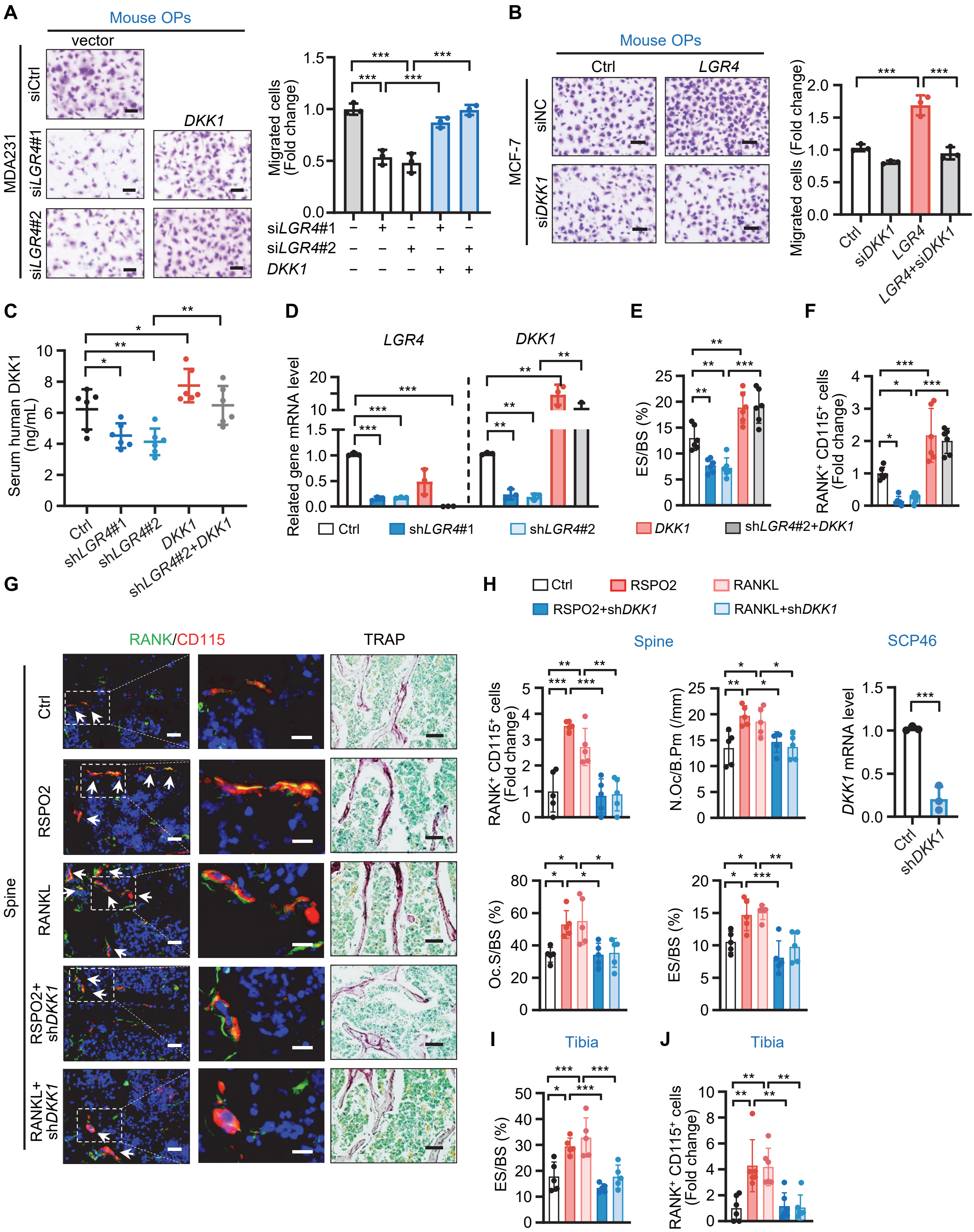

Supplemental Figure 7

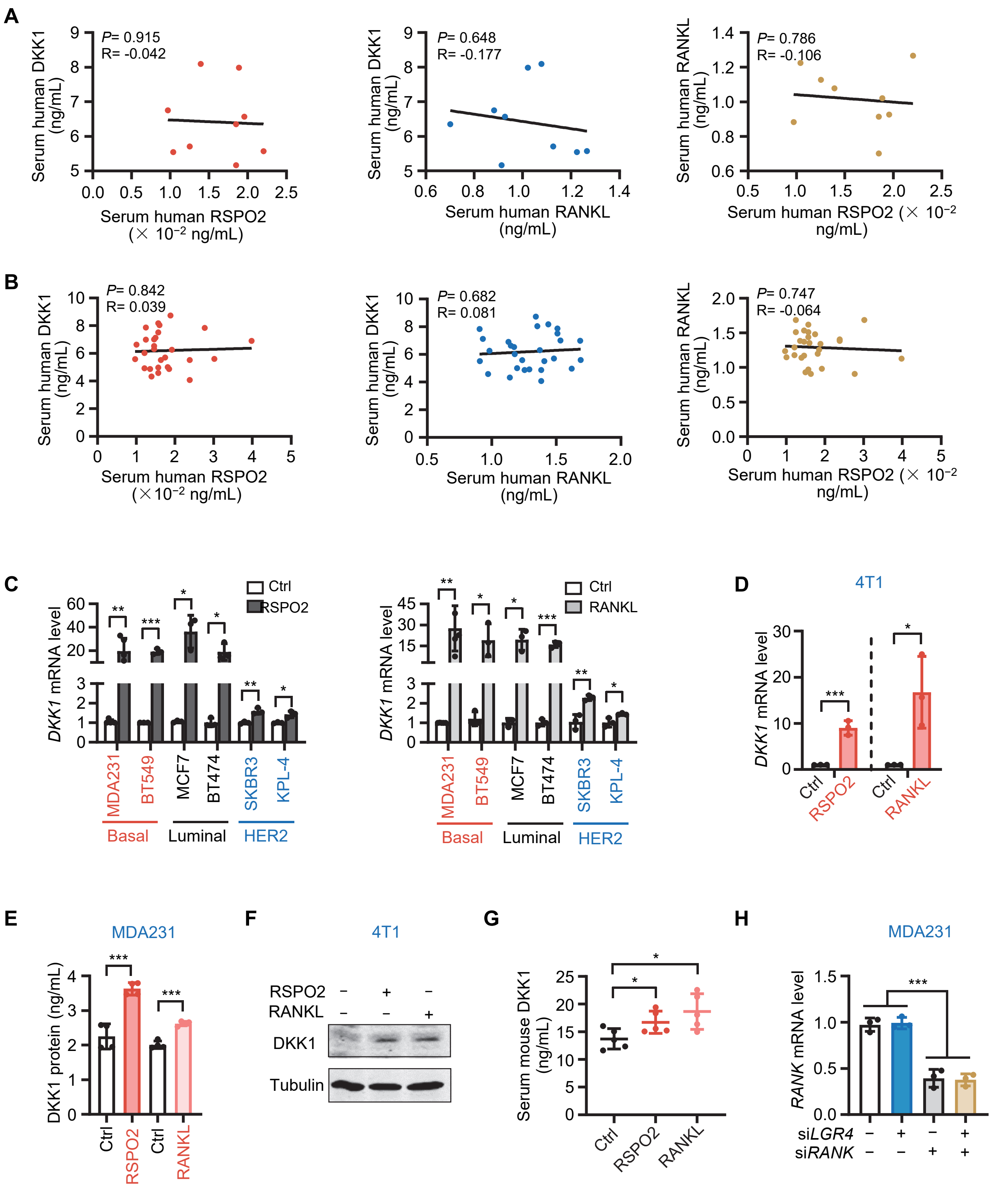

Supplemental Figure 8

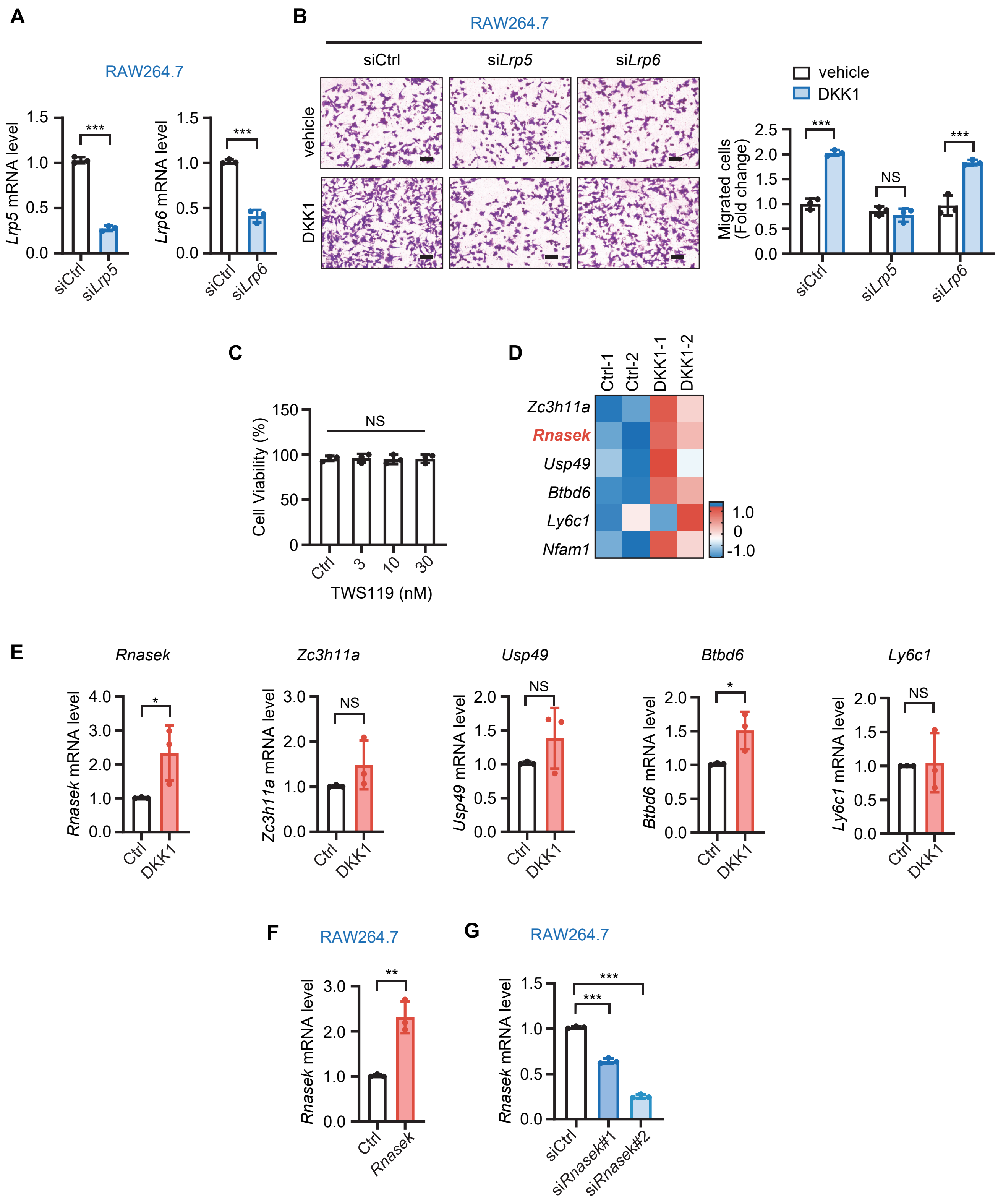

Supplemental Figure 9

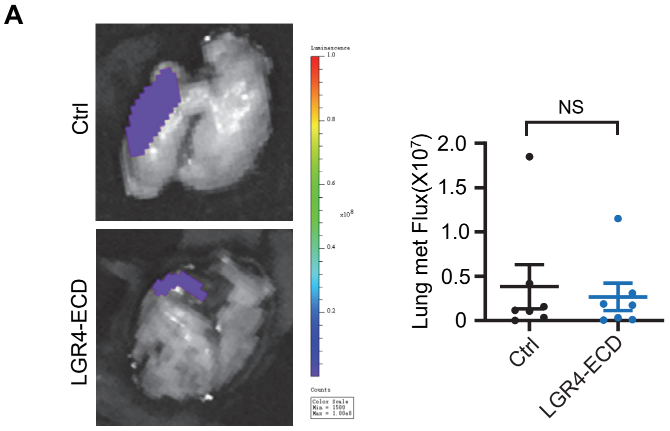

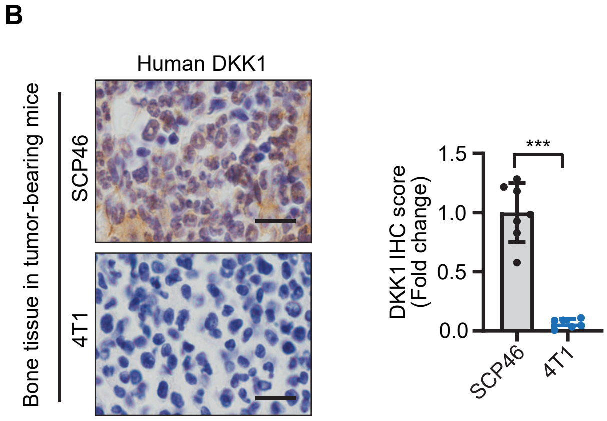

Supplemental Figure 10

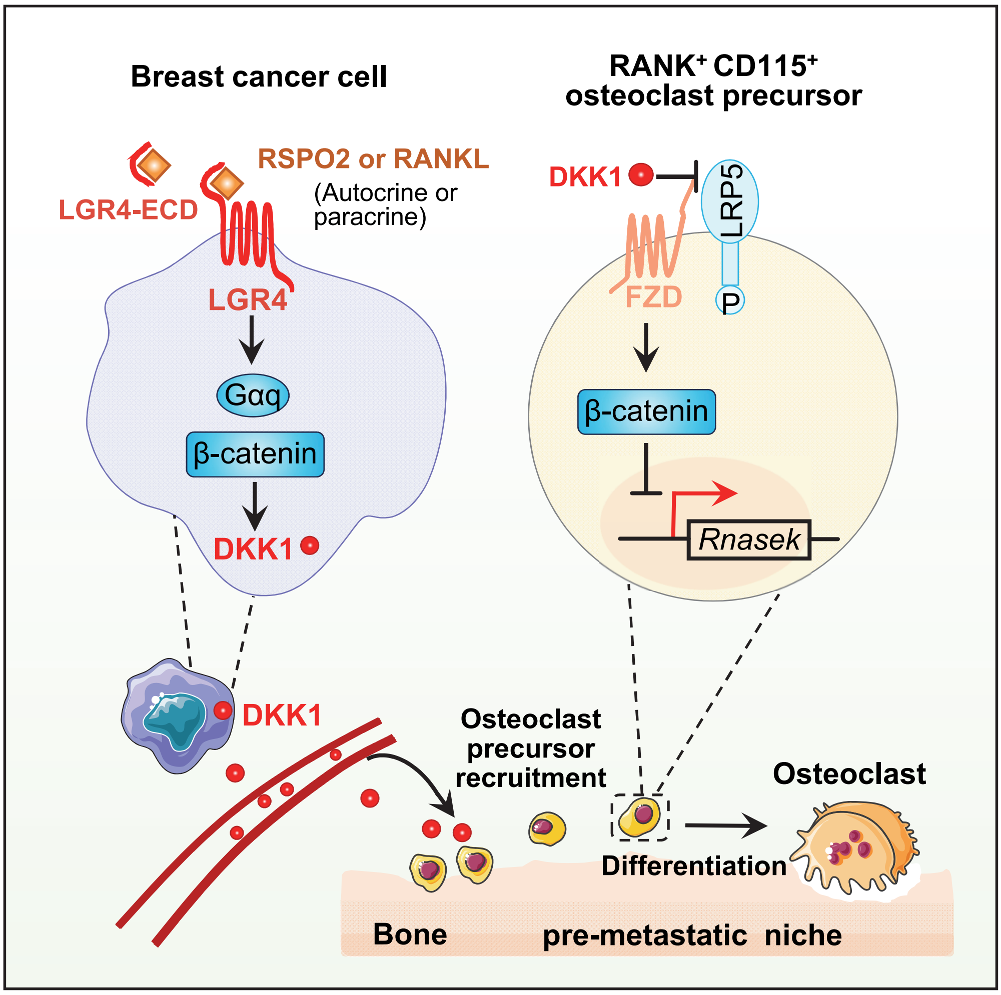

Supplementary Table 1. 86 GPCR agonists/ligands refer to Figure 1A

S/N	Agonist/Ligand	Receptor	S/N	Agonist/Ligand	Receptor	S/N	Agonist/Ligand	Receptor
1	Aripiprazole	5-HT1A Receptor	30	SKF-82958 (Hydrobromide)	Dopamine Receptor D1/D5	59	NS-304	IP Receptor
2	Vilazodone (Hydrochloride)	5-HT1A Receptor	31	rac-Rotigotine (Hydrochloride)	Dopamine Receptor D2	60	RSPO1	LGR4/LGR5/LGR6
3	Almotriptan (Malate)	5-HT1B/5-HT1D Receptor	32	Neuromedin N	Dopamine Receptor D2	61	RSPO2	LGR4/LGR5/LGR6
4	BRL 54443	5-HT1E/5-HT1F Receptor	33	Pramipexole (Dihydrochloride)	Dopamine Receptor D2S/D2L/D3/D4	62	RSPO3	LGR4/LGR5/LGR6
5	lumateperone (Tosylate)	5-HT2A Receptor	34	ST-836 (Hydrochloride)	Dopamine Receptor	63	RSPO4	LGR4/LGR5/LGR6
6	Lorcaserin (Hydrochloride)	5-HT2C Receptor	35	Dexpramipexole (Dihydrochloride)	Dopamine Receptor	64	NORRIN	LGR4/LGR5/LGR6
7	CP-809101 (Hydrochloride)	5-HT2C/5-HT2B/5-HT2A Receptor	36	Evatanepag	EP2 Receptor	65	VU0357017 (Hydrochloride)	M1/M2/M5 mAChR
8	Tegaserod (Maleate)	5-HT4 Receptor	37	Taprenepag	EP2 Receptor	66	Arecoline (Hydrobromide)	M3 mAChR
9	GR79236	Adenosine Receptor A1	38	Misoprostol	EP2/EP4 Receptor	67	VU0152100	M4 mAChR
10	CGS 21680 (Hydrochloride)	Adenosine Receptor A2A	39	PGE2	EP1/EP2/EP3/EP4 Receptor	68	Ramelteon	Melatonin Receptor 1/2
11	2-Cl-IB-MECA	Adenosine Receptor A3	40	Anamorelin	Ghrelin Receptor	69	Ro 67-7476	mGluR1
12	AdipoRon	Adiponectin Receptor 1/2	41	Betamethasone	Glucocorticoid Receptor	70	VU0364770	mGluR4
13	Scopine (Hydrochloride)	Adrenergic Receptor α1	42	Dexamethasone (Acetate)	Glucocorticoid Receptor	71	VU0357121	mGluR5
14	(R)-(-)-Phenylephrine (Hydrochloride)	Adrenergic Receptor α1A/α1B/α1D	43	Gonadorelin (Acetate)	GnRH	72	ML314	Neurotensin Receptor 1
15	UK 14304 (Tartrate)	Adrenergic Receptor α2A/α2B/α2C	44	Leuprolide (Acetate)	GnRH	73	Orexin 2 Receptor Agonist	Orexin Receptor 2
16	Dexmedetomidine (Hydrochloride)	Adrenergic Receptor α2A	45	Kynurenic Acid	GPR35	74	UDP	P2Y11/P2Y12 Receptor
17	Guanfacine (Hydrochloride)	Adrenergic Receptor α2A	46	AMG 837 (Calcium Hydrate)	GPR40	75	Diquafosol (Tetrasodium)	P2Y2 Receptor
18	Isoprenaline (Hydrochloride)	Adrenergic Receptor β(non-selective)	47	Sodium butyrate	GPR41/GPR43	76	ADP	P2Y6 Receptor
19	Norepinephrine (Bitartrate Monohydrate)	Adrenergic Receptor β1	48	Kisspeptin-10	GPR54	77	UDPN	P2Y14 Receptor
20	Ritodrine (Hydrochloride)	Adrenergic Receptor β2	49	OGERIN	GPR68	78	TRAP-6	Protease-Activated Receptor 1
21	Salbutamol (Hemisulfate)	Adrenergic Receptor β2(short-acting)	50	MBX-2982	GPR119	79	AC-55541	Protease-Activated Receptor 2
22	Indacaterol (Maleate)	Adrenergic Receptor β2(ultra-long-acting)	51	GSK137647A	GPR120	80	RANKL	RANK Receptor/LGR4
23	Mirabegron	Adrenergic Receptor β3	52	Type IV Collagen	GPR126	81	Siponimod	S1P1/S1P5 Receptor
24	WIN 55,212-2 (Mesylate)	CB1/CB2 Receptor	53	L-Lactic Acid	GPR132	82	Ozanimod	S1P1/S1P5 Receptor
25	Org 27569	CB1 Receptor	54	7α,25-OHC	GPR183	83	INT-777	TGR5
26	MDA 19	CB2 Receptor	55	Loratadine	Histamine Receptor H1	84	ADL-5859	δ-Opioid Receptor
27	JWH-133	CB2 Receptor	56	Histamine (Phosphate)	Histamine Receptor H1/H2/H3/H4	85	Loperamide (Hydrochloride)	μ-Opioid Receptor
28	SRT3190	CXCR2	57	Moxonidine	Imidazoline Receptor 1 (I1-R)	86	Trimebutine (Maleate)	μ-Opioid Receptor
29	Fenoldopam (Mesylate)	Dopamine Receptor D1	58	MRE-269	IP Receptor			

Supplementary Table 2. *LGR4* and *DKK1* expression value from GEO datasets of human breast cancer (MDA231) bone-tropic sublines (13 sublines)

Cell line / Gene	Parental MDA231	Weakly met SCP21	Weakly met SCP6	Weakly Bone met 2296	Weakly Bone met 2293	Weakly Bone met 2295	Strongly Bone met1833	Bone met SCP14	Bone met SCP25	Strongly Bone met 2269	Strongly Bone met 2268	Strongly Bone met 2274	Bone met SCP46
LGR4	1.061641	0.316673	0.448029	0.607448	0.792261	0.877346	0.938359	1.017849	1.024692	1.182555	1.233339	1.764152	2.170768
DKK1	1.120088	0.883666	0.804397	0.286618	0.290838	0.296921	1.232686	1.226288	1.232839	0.846338	1.287753	1.579341	1.630795

GEO DataSets: GSE14244 and GSE16554

Supplementary Table 4. Fold changes of 14 secreted WNT target genes in *LGR4* knock down SCP46 cells compared to shCtrl cells by qRT-PCR

Secreted Wnt targets	mRNA expression ratio	
	sh*LGR4*/shCtrl	STDEV
DKK1	**0.340**	**0.031**
DKK4	0.634	0.132
ADAM10	1.149	0.188
BMP4	1.164	0.144
EDN1	1.057	0.458
FGF18	0.572	0.101
FGFBP1	0.667	0.189
GAST	0.671	0.034
LAMC2	0.686	0.066
MMP7	1.017	0.084
PLAU	0.290	0.009
S100A4	0.427	0.011
TNC	0.469	0.092
VEGFA	0.276	0.027

Supplementary Table 5. 14 secreted Wnt target protein fraction of total (FOT) value in *LGR4* WT MDA468 cell medium compared to KO cell medium by proteomic profiling analysis

	FOT value	
Secreted Wnt targets	*LGR4* WT	*LGR4* KO
**DKK1**	**3.2**	**ND**
DKK4	ND	ND
ADAM10	2.7	1.8
BMP4	ND	ND
EDN1	ND	ND
FGF18	ND	ND
FGFBP1	109.4	85.0
GAST	ND	ND
LAMC2	ND	0.2
MMP7	ND	ND
PLAU	ND	ND
S100A4	36.2	90.9
TNC	ND	0.3
VEGFA	ND	ND

ND= not detected.

Supplementary Table 6. *LGR4* and *DKK1* expression (normalized to the expression in MCF7) in 8 different BCa cell lines by qRT-PCR

Cell line / Gene	T47D	MDA-MB-453	MCF7	MDA-MB-436	BT474	HS578T	BT549	MDA-MB-231
LGR4	0.18154	0.29918	1	1.16724	1.48433	2.56422	2.7644	3.48207
DKK1	0.2789	0.23749	1	3.82553	3.86976	8.11773	17.74853	18.63091

Supplementary Table 7. Primer sequences used in this study

Primer Name		Primer sequence
Human *LGR4*	F	ACCTGGAGACCTTAGACTTG
	R	CCACGAATGACTAGGGAATG
Human *RANK*	F	GGGAAAGCACTCACAGCTAATTTG
	R	GCACTGGCTTAAACTGTCATTCTCC
Human *DKK1*	F	CCTTGAACTCGGTTCTCAATTCC
	R	CAATGGTCTGGTACTTATTCCCG
Human *ADAM10*	F	ATGGGAGGTCAGTATGGGAATC
	R	ACTGCTCTTTTGGCACGCT
Human *BMP4*	F	ATGATTCCTGGTAACCGAATGC
	R	CCCCGTCTCAGGTATCAAACT
Human *DKK4*	F	ACGGACTGCAATACCAGAAAG
	R	CGTTCACACAGAGTGTCCCAG
Human *END1*	F	CTACTTCTGCCACCTGGACATC
	R	TCACGGTCTGTTGCCTTTGTGG
Human *FGF18*	F	ACGATGTGAGCCGTAAGCAGCT
	R	ACCGAAGGTGTCTGTCTCCACT
Human *FGFBP1*	F	TGGCAAACCAGAGGAAGACTGC
	R	GGAACCCGTTCTCTTTTGACCTC
Human *GAST*	F	ATGCAGCGACTATGTGTGTATG
	R	GCCCCTGTACCTAAGGGTG
Human *LAMC2*	F	GACAAACTGGTAATGGATTCCGC
	R	TTCTCTGTGCCGGTAAAAGCC
Human *MMP7*	F	TCGGAGGAGATGCTCACTTCGA
	R	GGATCAGAGGAATGTCCCATACC
Human *PLAU*	F	GGGAATGGTCACTTTTACCGAG
	R	GGGCATGGTACGTTTGCTG
Human *S100A4*	F	CAGAACTAAAGGAGCTGCTGACC
	R	CTTGGAAGTCCACCTCGTTGTC
Human *TNC*	F	ATGTCCTCCTGACAGCCGAGAA
	R	AGTCACGGTGAGGTTTTCCAGC
Human *VEGFA*	F	AGGGCAGAATCATCACGAAGT
	R	AGGGTCTCGATTGGATGGCA
Human *β-ACTIN*	F	GTACGCCAACACAGTGCTG
	R	CGTCATACTCCTGCTTGCTG
Mouse *Lgr4*	F	AAGATAACAGCCCCCAAGAC
	R	AGGCAGTGATGAACAAGACG
Mouse *Dkk1*	F	CAGTGCCACCTTGAACTCAGT
	R	CCGCCCTCATAGAGAACTCC
Mouse *Rnasek*	F	TGTTAATTGAGGACGTTCCCTTC
	R	GCGGCGATGAAACAGTTGTA
Mouse *Btdbd6*	F	GCCCACAAGTATGTCTTGGCT
	R	CAGGCTCCACATCGGGAATG
Mouse *Ly6c1*	F	GCAGTGCTACGAGTGCTATGG
	R	ACTGACGGGTCTTTAGTTTCCTT
Mouse *Usp49*	F	GGAGAATCTACGCTTGTGACCAG
	R	GGAGGACCTGAGGTAGTCTGTA
Mouse *Z3ch11a*	F	ATGCCTAATCAGGGAGAAGACT
	R	CACCTGTCGAAAACAACGACC
Mouse *Gapdh*	F	TGGCCTTCCGTGTTCCTAC
	R	GAGTTGCTGTTGAAGTCGCA
